# Supplementary material for: Finnish Retirement and Aging Study: a prospective cohort study
Source: BMJ Open. 2023 Dec 9;13(12):e076976. doi: 10.1136/bmjopen-2023-076976 (PMC10729264; doi:10.1136/bmjopen-2023-076976)
Supplement: Supplementary data [file bmjopen-2023-076976supp001.pdf]

Supplementary Table 1. Overview of the data collection methods in the Finnish Retirement and Aging study.

**Survey questionnaire**	
Sociodemographic characteristics	Gender, marital status
Health and wellbeing	Self-rated health, chronic conditions, vision, hearing, pain [1], height, weight, physical functioning (Short Form Health Survey (SF-36)) [1], falls, subjective memory complaints, depression [2,3], psychological wellbeing (General Health Questionnaire (GHQ)-12) [4], optimism and pessimism (Life Orientation test) [5], life satisfaction [6], sexual health (Female Sexual Function Index)[7], relationship happiness
Health behaviors	Sleep duration [8], sleep difficulties (Jenkins Sleep Problem Scale) [9], daytime tiredness [8], napping, sitting [10], leisure and commuting physical activity [11], smoking, alcohol consumption, diet (rhythm and content)
Social and living environment	Social networks [12], social participation [13], family caregiving, negative life events, neighborhood cohesion [14], health care use
Work and retirement	Workability [15], worktime mode, psychosocial stressors (job strain, work demands and control [16], worktime control [17], effort-reward imbalance [18], organizational justice [19]), physical work stressors (strenuous work, awkward working positions, repetitive work), plans for retirement timing, actual retirement date
**Device-based movement behavior measures**	
ActiGraph accelerometer (wActiSleep-BT and wGT3X-BT), wrist-worn	Total amount of physical activity as vector magnitude counts per minute (VM CPM) [20]. Time spent sedentary, in light physical activity, in moderate-to-vigorous physical activity, bouts (number and duration) of sedentary time, light physical activity and moderate-to-vigorous physical activity [21,22], sleep duration [22,23], sleep efficiency [23], bedtime and awakening time, ambient light [24]
Axivity accelerometer (AX3), thigh-worn	Time spent lying, sitting, standing, moving, walking slow, walking fast, stair walking, running, cycling and other physical activity. Number of steps and sit-to-stand transitions. Duration of lying, sitting and standing bouts [25,26]

Stenholm S, *et al. BMJ Open* 2023; 13:e076976. doi: 10.1136/bmjopen-2023-076976

Global Positioning System and accelerometer device (SenseDoc V.2.0), waist-worn	Time spent sedentary, in light physical activity and in moderate-to-vigorous physical activity in different locations and during travel [27-29].
**Clinical measurements**	
Blood samples	Fasting blood sample were drawn by venipuncture. All blood samples were aliquoted at the same day and then stored at -80°C. At the moment some samples have been analyzed at the laboratory of Turku University Hospital, Finland. Plasma cholesterol (total, low-density lipoprotein cholesterol, high-density lipoprotein cholesterol) and triglycerides were determined by enzymatic colorimetric tests (Cobas 8000 c702, Roche Diagnostics). C-reactive protein was determined by nephelometric method (BN ProSpex/Atellica Neph, Siemens Healthineers). Fasting glucose was determined by enzymatic reference method with hexokinase and fasting insulin was measured by ECLIA/Sandwich principle (Cobas 8000 e801, Roche Diagnostics).
Hair sample	A strand of hair was cut from a standardized area of the posterior vertex region of the head as close to the scalp as possible [30]. Hair samples were stored in foil in a dry place protected from light. Hair cortisol and cortisone were analyzed later using mass spectrometry (Technical University of Dresden, Germany).
Anthropometry and body composition	Weight, height, body mass index, waist circumference, body composition (Inbody 720, Biospace Co., Seoul, Korea) [31].
Cardiovascular measurements	Systolic and diastolic blood pressure from both arms and ankles (Microlife WatchBP Office Central, Microlife AG, Widnau, Switzerland) [32], ankle brachial index, 24-h ambulatory blood pressure (Microlife WatchBP O3 Monitor, Microlife AG, Widnau, Switzerland) [32], carotid-femoral Pulse Wave Velocity (SphygmoCor PVx with MM3 electronic module and Millar tonometer) [33].
Physical fitness	VO2peak measured with indirect submaximal bicycle ergometer test with Ergoselect 100 K (Ergoline, Bitz, Germany) according to the American College of Sports Medicine guidelines [31,34], modified push-up test [31,35], sit-up test [35].

Physical functioning	Maximal and normal walking speed over 4 meters [36], Short Physical Performance Battery [37], 1-leg standing balance [38], hand grip strength from the dominant hand (Jamar dynamometer).
Cognitive functioning	Cambridge Neuropsychological Test Automated Battery (CANTAB®), computerized test battery covering multiple cognitive domains including learning and memory, working memory, information processing, and reaction time [39], Trail Making Test [40], Mini Mental State Examination and verbal recall from the Consortium to Establish a Registry for Alzheimer's Disease (CERAD) [41].
Self-reported medication use	Anatomical Therapeutic Chemical (ATC) Classification codes for each reported drug.
**Register data**	
Sociodemographic characteristics	Date of birth, sex, occupational code, employment history (Pension insurance institute Keva)
National health registers	Primary diagnosis and date of hospital discharge from years 1987-2019 (Care Register for Health Care maintained by the Finnish Institute for Health and Welfare); medication purchase, special reimbursement, rehabilitation, sick-leave days from years 1994-2019 (Registers of the Social Insurance Institution of Finland); type of cancer, date of diagnosis years 1953-2019 (Cancer register)
Occupational injuries	Types and causes of workplace and commuting injuries from years 2000-2019 (the Federation of Accident Insurance Institutions)
Residential environment characteristics	Home address; socioeconomic disadvantage: median household income, low educational attainment (percentage of people over 18 years old with low education) and unemployment rate from each 250x250m square [42]; Greenness in the neighbourhood calculated as Normalised Difference Vegetation Index (NDVI) for each map grip from a satellite image composite using Google Earth Engine [42]. Mean, maximum and minimum temperature and humidity data from >500 weather stations over Finland interpolated to a spatial resolution of 1x1km^2 to measure continuous daily gridded time series of daily temperature.

References

1. Hayes R, Sherbourne C, Mazel R. The RAND 36-item Health Survey 1.0. *Health Economics* 1993; **2**: 217–27.
2. Arroll B, Khin N, Kerse N. Screening for depression in primary care with two verbally asked questions: cross sectional study. *Bmj* 2003; **327**(7424): 1144-6.
3. Beck AT, Ward CH, Mendelson M, Mock J, Erbaugh J. An inventory for measuring depression. *Arch Gen Psychiatry* 1961; **4**: 561-71.
4. Goldberg DP. The detection of psychiatric illness by questionnaire. Oxford: Oxford university press; 1972.
5. Kronström K, Karlsson H, Nabi H, et al. Optimism and pessimism as predictors of work disability with a diagnosis of depression: a prospective cohort study of onset and recovery. *J Affect Disord* 2011; **130**(1-2): 294-9.
6. Koivumaa-Honkanen H, Honkanen R, Viinamäki H, Heikkilä K, Kaprio J, Koskenvuo M. Self-reported life satisfaction and 20-year mortality in healthy Finnish adults. *Am J Epidemiol* 2000; **152**(10): 983-91.
7. Rosen R, Brown C, Heiman J, et al. The Female Sexual Function Index (FSFI): a multidimensional self-report instrument for the assessment of female sexual function. *Journal of sex & marital therapy* 2000; **26**(2): 191-208.
8. Myllyntausta S, Salo P, Kronholm E, et al. Does removal of work stress explain improved sleep following retirement? The Finnish Retirement and Aging study. *Sleep* 2019; **42**(8): zsz109.
9. Jenkins CD, Stanton BA, Niemcryk SJ, Rose RM. A scale for the estimation of sleep problems in clinical research. *Journal of clinical epidemiology* 1988; **41**(4): 313-21.
10. Leskinen T, Pulakka A, Heinonen OJ, et al. Changes in non-occupational sedentary behaviours across the retirement transition: the Finnish Retirement and Aging (FIREA) study. *J Epidemiol Community Health* 2018; **72**(8): 695-701.
11. Kujala UM, Kaprio J, Sarna S, Koskenvuo M. Relationship of leisure-time physical activity and mortality: the Finnish twin cohort. *Journal of American Medical Association* 1998; **279**(6): 440-4.
12. Antonucci T. Measuring social support networks: Hierarchical mapping technique. *Generations: Journal of the American Society on Aging* 1986; **10**(4): 10-2.
13. Kauppi M, Prakash KC, Virtanen M, et al. Social relationships as predictors of extended employment beyond the pensionable age: a cohort study. *Eur J Ageing* 2021; **18**(4): 491-501.
14. Lahdenperä M, Virtanen M, Myllyntausta S, Pentti J, Vahtera J, Stenholm S. Psychological Distress During the Retirement Transition and the Role of Psychosocial Working Conditions and Social Living Environment. *J Gerontol B Psychol Sci Soc Sci* 2022; **77**(1): 135-48.
15. Ilmarinen J, Tuomi K, Klockars M. Changes in the work ability of active employees over an 11-year period. *Scand J Work Environ Health* 1997; **23 Suppl 1**: 49-57.
16. Karasek R, Brisson C, Kawakami N, Houtman I, Bongers P, Amick B. The Job Content Questionnaire (JCQ): an instrument for internationally comparative assessments of psychosocial job characteristics. *J Occup Health Psychol* 1998; **3**(4): 322-55.
17. Ala-Mursula L, Vahtera J, Linna A, Pentti J, Kivimäki M. Employee worktime control moderates the effects of job strain and effort-reward imbalance on sickness absence: the 10-town study. *J Epidemiol Community Health* 2005; **59**(10): 851-7.
18. Siegrist J, Wege N, Pühlhofer F, Wahrendorf M. A short generic measure of work stress in the era of globalization: effort-reward imbalance. *International archives of occupational and environmental health* 2009; **82**(8): 1005-13.

19. Elovainio M, Kivimäki M, Vahtera J. Organizational justice: evidence of a new psychosocial predictor of health. *Am J Public Health* 2002; **92**(1): 105-8.
20. Pulakka A, Leskinen T, Koster A, Pentti J, Vahtera J, Stenholm S. Daily physical activity patterns among aging workers: the Finnish Retirement and Aging Study (FIREA). *Occup Environ Med* 2019; **76**(1): 33-9.
21. Suorsa K, Leskinen T, Pasanen J, et al. Changes in the 24-h movement behaviors during the transition to retirement: compositional data analysis. *Int J Behav Nutr Phys Act* 2022; **19**(1): 121.
22. Migueles JH, Rowlands AV, Huber F, Sabia S, van Hees VT. GGIR: A research Community–Driven open source R package for generating physical activity and sleep outcomes from multi-day raw accelerometer data. *Journal for the Measurement of Physical Behaviour* 2019; **2**(3): 188-96.
23. Myllyntausta S, Pulakka A, Salo P, et al. Changes in accelerometer-measured sleep during the transition to retirement: the Finnish Retirement and Aging (FIREA) study. *Sleep* 2020; **43**(7): zsz318.
24. Flynn JI, Coe DP, Larsen CA, Rider BC, Conger SA, Bassett DR, Jr. Detecting indoor and outdoor environments using the ActiGraph GT3X+ light sensor in children. *Med Sci Sports Exerc* 2014; **46**(1): 201-6.
25. Skotte J, Korshoj M, Kristiansen J, Hanisch C, Holtermann A. Detection of physical activity types using triaxial accelerometers. *J Phys Act Health* 2014; **11**(1): 76-84.
26. Stemland I, Ingebrigtsen J, Christiansen CS, et al. Validity of the Acti4 method for detection of physical activity types in free-living settings: comparison with video analysis. *Ergonomics* 2015; **58**(6): 953-65.
27. Thierry B, Chaix B, Kestens Y. Detecting activity locations from raw GPS data: a novel kernel-based algorithm. *International journal of health geographics* 2013; **12**: 14.
28. Pasanen S, Halonen JI, Pulakka A, et al. Contexts of sedentary time and physical activity among ageing workers and recent retirees: cross-sectional GPS and accelerometer study. *BMJ Open* 2021; **11**(5): e042600.
29. Pasanen S, Halonen JI, Suorsa K, et al. Does work-related and commuting physical activity predict changes in physical activity and sedentary behavior during the transition to retirement? GPS and accelerometer study. *Health Place* 2023; **81**: 103025.
30. Greff MJE, Levine JM, Abuzgaia AM, Elzagallaai AA, Rieder MJ, van Uum SHM. Hair cortisol analysis: An update on methodological considerations and clinical applications. *Clinical biochemistry* 2019; **63**: 1-9.
31. Stenholm S, Pulakka A, Leskinen T, et al. Daily Physical Activity Patterns and Their Association With Health-Related Physical Fitness Among Aging Workers-The Finnish Retirement and Aging Study. *J Gerontol A Biol Sci Med Sci* 2021; **76**(7): 1242-50.
32. Karelius S, Vahtera J, Suorsa K, et al. Changes in ambulatory blood pressure during the transition to retirement. *J Hypertens* 2023; **41**(1): 187-93.
33. Lindroos AS, Johansson JK, Puukka PJ, et al. The association between home vs. ambulatory night-time blood pressure and end-organ damage in the general population. *J Hypertens* 2016; **34**(9): 1730-7.
34. American College of Sports Medicine. ACSM's Guidelines for Exercise Testing and Prescription. 9th ed. Baltimore: Wolters Kluwer, Lippincott Williams & Wilkins; 2014.
35. Suorsa K, Mattila VM, Leskinen T, et al. Work ability and physical fitness among aging workers: the Finnish Retirement and Aging Study. *Eur J Ageing* 2022; **19**(4): 1301-10.

36. Guralnik JM, Ferrucci L, Simonsick EM, Salive ME, Wallace RB. Lower-extremity function in persons over the age of 70 years as a predictor of subsequent disability. *New England Journal of Medicine* 1995; **332**: 556-61.
37. Guralnik JM, Simonsick EM, Ferrucci L, et al. A Short Physical Performance Battery Assessing Lower Extremity Function: Association With Self-Reported Disability and Prediction of Mortality and Nursing Home Admission. *J Gerontol* 1994; **49**(2): M85-M94.
38. Suni JH, Oja P, Miilunpalo SI, Pasanen ME, Vuori IM, Bos K. Health-related fitness test battery for adults: associations with perceived health, mobility, and back function and symptoms. *Arch Phys Med Rehabil* 1998; **79**(5): 559-69.
39. Teräs T, Rovio S, Spira AP, et al. Associations of accelerometer-based sleep duration and self-reported sleep difficulties with cognitive function in late mid-life: the Finnish Retirement and Aging Study. *Sleep Med* 2020; **68**: 42-9.
40. Reitan RM. Trail Making Test: Manual for Administration and Scoring. Tuscon, AZ: Reitan Neuropsychology Laboratory, 1992.
41. Morris JC, Heyman A, Mohs RC, et al. The consortium to establish a registry for Alzheimer´s disease (CERAD). Part 1. Clinical and neuropsychological assessement of Alzheimer´s disease. *Neurology* 1989; **39**: 1159-65.
42. Halonen JI, Pulakka A, Pentti J, et al. Cross-sectional associations of neighbourhood socioeconomic disadvantage and greenness with accelerometer-measured leisure-time physical activity in a cohort of ageing workers. *BMJ Open* 2020; **10**(8): e038673.
