# Supplementary material for: Finnish Retirement and Aging Study: a prospective cohort study
Source: BMJ Open. 2023 Dec 9;13(12):e076976. doi: 10.1136/bmjopen-2023-076976 (PMC10729264; doi:10.1136/bmjopen-2023-076976)
Supplement: Author's manuscript [file bmjopen-2023-076976.draft_revisions.pdf]

BMJ Open is committed to open peer review. As part of this commitment we make the peer review history of every article we publish publicly available.

When an article is published we post the peer reviewers' comments and the authors' responses online. We also post the versions of the paper that were used during peer review. These are the versions that the peer review comments apply to.

The versions of the paper that follow are the versions that were submitted during the peer review process. They are not the versions of record or the final published versions. They should not be cited or distributed as the published version of this manuscript.

BMJ Open is an open access journal and the full, final, typeset and author-corrected version of record of the manuscript is available on our site with no access controls, subscription charges or pay-per-view fees (http://bmjopen.bmj.com).

If you have any questions on BMJ Open's open peer review process please email info.bmjopen@bmj.com

BMJ Open

# Cohort Profile: Finnish Retirement and Aging study (FIREA), a prospective cohort study

| | |
|---|---|
| Journal: | *BMJ Open* |
| Manuscript ID | bmjopen-2023-076976 |
| Article Type: | Cohort profile |
| Date Submitted by the Author: | 22-Jun-2023 |
| Complete List of Authors: | Stenholm, Sari; University of Turku, Department of Public Health; Turku University Hospital<br>Suorsa, Kristin; University of Turku, Department of Public Health<br>Leskinen, Tuija; Turun Yliopisto<br>Myllyntausta, Saana; Turun Yliopisto, Department of Psychology and Speech-Language Pathology<br>Pulakka, Anna; University of Oulu; Finnish Institute for Health and Welfare<br>Pentti, Jaana; University of Turku, Department of Public Health<br>Vahtera, Jussi; Turun Yliopisto, Dep Public Health; Finnish Institute of Occupational Health, |
| Keywords: | Aging, OCCUPATIONAL & INDUSTRIAL MEDICINE, PREVENTIVE MEDICINE, PUBLIC HEALTH |
| | |

SCHOLARONE™
Manuscripts

*I, the Submitting Author has the right to grant and does grant on behalf of all authors of the Work (as defined in the below author licence), an exclusive licence and/or a non-exclusive licence for contributions from authors who are: i) UK Crown employees; ii) where BMJ has agreed a CC-BY licence shall apply, and/or iii) in accordance with the terms applicable for US Federal Government officers or employees acting as part of their official duties; on a worldwide, perpetual, irrevocable, royalty-free basis to BMJ Publishing Group Ltd ("BMJ") its licensees and where the relevant Journal is co-owned by BMJ to the co-owners of the Journal, to publish the Work in this journal and any other BMJ products and to exploit all rights, as set out in our licence.*

*The Submitting Author accepts and understands that any supply made under these terms is made by BMJ to the Submitting Author unless you are acting as an employee on behalf of your employer or a postgraduate student of an affiliated institution which is paying any applicable article publishing charge ("APC") for Open Access articles. Where the Submitting Author wishes to make the Work available on an Open Access basis (and intends to pay the relevant APC), the terms of reuse of such Open Access shall be governed by a Creative Commons licence – details of these licences and which Creative Commons licence will apply to this Work are set out in our licence referred to above.*

*Other than as permitted in any relevant BMJ Author's Self Archiving Policies, I confirm this Work has not been accepted for publication elsewhere, is not being considered for publication elsewhere and does not duplicate material already published. I confirm all authors consent to publication of this Work and authorise the granting of this licence.*

For peer review only

# Cohort Profile: Finnish Retirement and Aging study (FIREA), a prospective cohort study

Sari Stenholm[1,2,3], Kristin Suorsa[1,2], Tuija Leskinen[1,2], Saana Myllyntausta[4], Anna Pulakka[5,6], Jaana Pentti[1,2,7], Jussi Vahtera[1,2]

[1] Department of Public Health, University of Turku and Turku University Hospital, Turku, Finland;

[2] Centre for Population Health Research, University of Turku and Turku University Hospital; Turku, Finland;

[3] Research Services, Turku University Hospital and University of Turku, Finland

[4] Department of Psychology and Speech-Language Pathology, University of Turku, Turku, Finland

[5] Research Unit of Population Health, Faculty of Medicine, University of Oulu, Oulu, Finland

[6] Population Health Unit, Finnish Institute for Health and Welfare, Helsinki, Finland

[7] Clinicum, Faculty of Medicine, University of Helsinki, Helsinki, Finland

Correspondence:

Prof. Sari Stenholm, Department of Public Health, University of Turku, Finland

Postal address: Public Health, University of Turku, FI-20014 University of Turku, Finland

Email: sari.stenholm@utu.fi ; Telephone: +358 50 465 1745

For peer review only

## ABSTRACT

**Purpose:** The Finnish Retirement and Aging study (FIREA) was set up to study changes in health behavioral and cardiometabolic risk factors across retirement transition, and to examine the long-term consequences of work and retirement on health and functioning with advancing age.

**Participants:** Public sector workers whose estimated statutory retirement date was in 2014–2019 were invited to participate by sending them a questionnaire 18 months prior to their estimated retirement date. In the first phase of the FIREA study, participants were followed up with annual surveys, accelerometer and clinical measurements during retirement transition into post-retirement years. The FIREA survey cohort includes 6,783 participants, of which 908 belong also to the activity sub-study, and 290 to the clinical sub-study.

**Findings to date:** Collected data includes survey measures about health, lifestyle factors, psychosocial distress, work-related factors as well as retirement intentions. Accelerometer and GPS devices are used to measure 24-hour movement behaviors. Clinical examination includes blood and hair sample, measurements of anthropometry, cardiovascular function, physical fitness, physical and cognitive function. Our results suggest that in general retirement transition seems to have beneficial influence on health behaviors as well as on physical and mental health, but there are large individual differences, and certain behaviors such as sedentariness tend increase especially among those retiring from manual occupations.

**Future plans:** The second phase of the FIREA study will be conducted during 2023–2024, when participants are 70 years old. The FIREA study welcomes research collaboration proposals that fall within the general aims of the project.


For peer review only

## Strengths and limitations of this study

- The unique feature of the FIREA study is that the data collection was targeted around estimated statutory retirement date and it continued annually from final working years into actual retirement.
- In addition to survey measures participants have been followed with accelerometer and clinical measurements, which are sensitive to capture subtle changes in movement behaviors and cardiometabolic risk markers.
- The extensive Finnish national registers offer a possibility to complement collected data with information on participants' health, use of health care services as well as on neighborhood characteristics.
- The participation rate in the first phase of the FIREA study has been good, especially among those who participated in the activity (≥88% across measurements years) and the clinical sub-study (≥92% across measurements years).
- The main limitation for the FIREA study is a healthy worker effect as the inclusion criteria include participation in the working life after the age of 60. Those retired due to illness, disability or unemployed are not included in the study population, which limits the generalizability of the results especially related to health-related outcomes.

For peer review only

## Introduction

The Finnish Retirement and Aging study (FIREA) was established in 2013 to understand in detail the short and long term effects of work and retirement, an important life transition in late midlife, on people's health behaviors and health. Specifically, the FIREA study was initiated 1) to examine changes in health behaviors, cardiometabolic risk factors, and health indicators during retirement transition by following older workers from final working years to full-time retirement; 2) to study individual- and work-related factors that predict working life participation in people aged 60 years and older; and 3) to examine long-term consequences of work and retirement on health and functioning with an advancing age.

Transitioning from working life into retirement brings flexibility to daily time use, while work-related stressors are also removed, which may have extensive impact on health behaviors and social activities and consequently on health and well-being. Although several studies on retirement and health had been published at the time FIREA was initiated [1,2], only few studies had been able to follow individuals through retirement transition. These studies had reported changes in health behaviors, such as increased physical activity and improved sleep [3-7]. However, the previous evidence, coming mostly from non-generalizable study populations, was contrasting, and information on health and health behaviors was based on self-reported measures. To identify subtle changes in health behaviors and clinical risk markers, and to overcome measurement bias related to survey measures, the FIREA study took one step further by establishing annual device-based and clinical measurements for older workers close to their estimated retirement data. These measurements have been complemented with annual surveys, including questions about lifestyle factors, physical and mental health, work and social factors, as well as with register-based information on participants' living environment and linkage to


For peer review only - http://bmjopen.bmj.com/site/about/guidelines.xhtml

national health registries. The aim is to continue following up the study participants until old age.

## Cohort description

*Study population and data collection*

The eligible study population of the FIREA cohort comprised of public sector employees who were working in any town in Southwest Finland or in any of the a priori selected 9 towns or five hospital districts around Finland in 2012 and whose estimated statutory retirement date was between 2012 and 2025 (N=47,581). This register cohort was acquired from the pension insurance institute for the municipal sector in Finland (Keva), and included information about workers' date of birth, work history and estimated retirement date. Individuals whose estimated retirement date was between 2014 and 2019, were invited to participate in the FIREA study by sending them a questionnaire 18 months prior to their estimated retirement date (N=10,629). The data collection started in the fall 2013. In the first phase of the FIREA study, questionnaires were sent once a year with an aim to gather data from at least two time points before and two time points after the transition to statutory retirement, up to seven times in total. In total, 6,783 participants had responded to at least one questionnaire by the end of 2018. Flow chart of the FIREA study is shown in Figure 1, and schematic overview of the data collection in Figure 2.

In 2014, a FIREA activity sub-study was launched and Finnish-speaking FIREA survey cohort participants who were still working and had an estimated statutory retirement date between 2016 and 2019 were invited to participate (n=2,663). Of them, 908 consented to participate and were followed via

For peer review only

accelerometer measurements and survey questionnaires annually, at the same time of the year, up to five times in total.

One year later in 2015, a FIREA clinical sub-study was initiated. Finnish-speaking survey cohort participants who were still working, had an estimated statutory retirement date between 2017 and 2019 and who lived in the Southwest Finland were invited to participate (n=773). Of them, 290 consented to participate in annual clinical examination at the University of Turku. Up to five annual follow-up visits were conducted until the participants had retired.

Due to COVID-19 pandemic and consequent national restrictions, the data collection for activity and clinical sub-study were temporarily discontinued between March and June in 2020. However, this influenced only a small proportion of the participants, since majority of the participants had already retired and completed the first phase of the data collection before 2020.

Table 1 provides comparison between the participants in the survey cohort, the activity sub-study, the clinical sub-study, and the non-participants. In comparison to the FIREA participants, the proportions of men and manual workers were greater among the non-participants. Participants in the survey cohort, the activity sub-study, and the clinical sub-study did not differ from each other in terms of age, sex, and marital status, but the proportions of manual workers and current smokers were greater in the survey cohort compared to activity sub-study and clinical sub-study participants. Self-reported physical activity was highest and self-rated health better among the clinical sub-study participants and lowest and less optimal, respectively, in the survey cohort.

In the first phase of the FIREA study (2013–2022), the data collection took place annually, always at the same time of the year. On average, the participants provided survey data on 3.6 measurement points (range 1–7, SD 1.4). The mean

For peer review only

number of annual measurements in the activity sub-study was 3.7 (range 1–5, SD 0.7) and in the clinical sub-study 3.0 (range 1–5, SD 0.6).

Of the initial survey cohort, 58% have both pre- and post-retirement data (n=3,948), because many of the initial cohort had already retired when they were contacted for the first time. The actual retirement date was obtained from the survey and this information was used to identify pre- and post-retirement measurements. Respective proportions for the pre- and post-retirement measurements are 83% for the activity sub-study and 88% for the clinical sub-study. The higher proportions compared to the survey cohort reflect the fact that only those who were still working were invited to participate in these sub-studies.

The second phase of the FIREA study will take place in 2023–2025, when participants reach the age of 70 years. All participants who have participated to at least one survey, accelerometer measurement, or clinical examination visit will be invited to the respective follow-up measurements. The long-term aim is to conduct subsequent follow-ups every five years and follow participants into old age.

*Measurements*

*Survey measurements.* In the first phase of the FIREA study, annual surveys included questions about health, chronic diseases, physical functioning, memory, lifestyle factors, psychosocial distress, social networks, work-related physical and psychological stressors as well as retirement intentions and actual retirement date. Details of the survey instruments are provided in Supplement Table 1. In addition to the multiple-choice questions, the survey included open-end questions regarding positive expectations and fears about retirement for those who were still working,


For peer review only - http://bmjopen.bmj.com/site/about/guidelines.xhtml

and about positive and negative consequences of retirement for those who had retired.

*Activity sub-study measurements.* Participants' 24-hour movement behaviors were measured using wrist-worn triaxial ActiGraph wActiSleep-BT and wGT3X-BT accelerometers (ActiGraph, Pensacola, Florida, US), which were mailed to the participants. Participants were asked to wear the accelerometer on their non-dominant wrist for seven consecutive days and six consecutive nights at all times and fill in a diary of their workdays and days off, daily bedtimes and modes of commute. Details of the FIREA accelerometer measurement protocol has been described earlier [8]. Moreover, accelerometer data reduction procedures have been described in detail earlier [8-11].

*Clinical sub-study measurements.* Clinical examination visit included collection of a fasting blood sample and a hair sample, measurements of anthropometry, body composition, cardiovascular function, physical and cognitive function. Measurements were conducted by trained study nurses. During the clinical visit, the participants also responded to depression and dietary questionnaires and they were given a sexual health questionnaire to be filled at home. Details of these measurements are provided in Supplement Table 1.

Prior to the clinical examination, the participants received a wrist-worn triaxial ActiGraph wActiSleep-BT accelerometer via mail and were instructed to wear it continuously on their non-dominant wrist for 24 hours per day for at least seven days and nights, including at least two workdays and two days off. Detailed description about the wrist-worn accelerometer data collection and data reduction procedures applied to the clinical sub-study have been provided elsewhere [12].

During the clinical examination visit, the participants were provided two additional devices to measure their movement behavior and the study nurse



For peer review only - http://bmjopen.bmj.com/site/about/guidelines.xhtml

instructed the use of these devices. While using the devices the participants were asked to fill in a daily diary, in which they provided information on their workdays and days off, and on their daily bedtimes and modes of commute. The study nurse fastened a triaxial Axivity AX3 accelerometer (Axivity Ltd., Newcastle, UK) to the participants' right thigh using adhesive waterproof film dressing. Participants were asked to wear the accelerometer for at least two workdays and two days off at all times when still working, and a minimum of any four days when retired. Fastening the accelerometer to thigh enables identification of postures such as sitting and standing and types of physical activity such as walking, running, and cycling based on thigh angle and acceleration [13,14]. Detailed measurement protocol and accelerometer data reduction procedures for thigh-worn accelerometers have been described elsewhere [15]. In addition, participants received a waist-worn SenseDoc 2.0 device (Mobysens Technologies Inc, Canada), which includes a Global Positioning System (GPS) sensor and a tri-axial accelerometer [16]. The combined GPS and accelerometer data provide information about intensity of the physical activity, but also about activity contexts and locations that is home, transportation, and allows linking environmental characteristics to these locations. Participants were requested to wear the device during waking hours for at least two workdays and two days off when still working, and a minimum of any four days when retired. Details of the measurements and data processing have been described earlier [17,18].

Within one month from the initial clinical examination visit, physical fitness tests were performed by an experienced exercise physiologist and pulse wave velocity measurement was conducted by study nurse. Details of these measurements are provided in Supplement Table 1.

*Linkage to register data.* Data collected from the study participants have been complemented with national register data and the linkage is conducted by using the



For peer review only - http://bmjopen.bmj.com/site/about/guidelines.xhtml

national personal identification numbers (unique number assigned to all Finnish residents). Health-related outcomes are available until the end of 2019 from the hospital discharge information recorded by the Finnish Institute for Health and Welfare and the Cancer Register. In addition, medication purchase, special reimbursement, rehabilitation, sick-leave days are available from the registers of the Social Insurance Institution of Finland, and types and causes of workplace and commuting injuries from the Federation of Accident Insurance Institutions. Details of the register data are provided in Supplement Table 1.

Several indicators of the neighbourhood characteristics, such as a standardized index for socioeconomic disadvantage, degree of greenness, and daily temperature in the neighbourhood, have been derived by using information on participants' geocoded residential addresses from the Population Register Centre of Finland and these data were positioned to the Statistics Finland Grid Database, a 250×250m map grid that covers the whole country [19]. Details of these neighborhood-level indicators are provided in Supplement Table 1.

*Patient and public involvement*

Study population or the general public were not involved in the planning, design or conduction of the study.

## Findings to date

*Movement behaviors*

One of the focuses of the FIREA study has been to understand how movement behaviors change when people transition from full time work to retirement. By using annually collected wrist-accelerometer data and by addressing differences between sex and occupations, we have shown that women retiring from manual occupations



For peer review only - http://bmjopen.bmj.com/site/about/guidelines.xhtml

decrease their physically activity, whereas men from non-manual occupations slightly increase physical activity after retirement [20]. Despite the declining trend among women, they are still more active than men both before and after retirement. Our results also suggest that sleep duration and sedentary time, especially among women retiring from manual occupations, increase after retirement [9,10,21,22]. More recently, we have applied compositional data analysis (CoDA), which allows examination of concomitant changes of the 24-h movement behaviors, that is physical activity, sedentary time, and sleep [11]. Our findings suggest that retirement decreases the proportion of time spent in active behaviors, especially moderate-to-vigorous physical activity, in relative to time spent in passive behaviors, such as sedentary time and sleep. These findings highlight that retirement is an important time period in late midlife for paying special attention to activity behavior and develop new daily routines, which can replace work- and commuting-related activities and involve physical activity in different forms. With advancing age, the importance of physically active lifestyle is emphasized, because it helps not only to prevent and treat non-communicable diseases, such as cardiovascular disease, type 2 diabetes, and various cancers [23], but it also helps to maintain physical functioning [24] and improve mental well-being [25].

*Health and mental well-being*

In addition to movement behaviors, we have also been interested in retirement-induced changes in various health and wellbeing indicators. We have shown that psychological distress decreases drastically after retirement and especially among those with poorer psychosocial working conditions and social living environment [26]. Similarly, life satisfaction increases, most prominently among women, those with suboptimal health, and those living without a spouse [27]. Previous studies have

For peer review only

reported both improvement and decline in self-reported health after retirement [4,28-30], which was also confirmed by our findings. However, by using latent trajectory analysis to identify groups of individuals who show similar developmental trajectories over time, we found that a large majority of participants actually maintain their perceived health status during retirement transition and smaller subgroups of people show improvement or decline in perceived health [31]. Those whose health perception declined during the retirement transition, had lower occupational status, physically strenuous work, and job strain. Overall, our findings suggest that people with physically or psychosocially demanding work may improve their psychological wellbeing after retirement, but this is not necessarily reflected in their health perception. Our future work with the extended follow-up will reveal more about the long-term development of health, morbidity as well as physical and cognitive functioning in the post-retirement years.

*Predictors of extended employment*

By using the information about timing of retirement, we have examined how factors related to health, work, as well as family and social relations predict retirement timing. We have observed that those who have better health and work ability [32], good worktime control [33], and full-time working spouse [34] are more likely to extend their employment beyond the pensionable age compared to those who retire at their state pension age or earlier. We have also shown that men are more likely to extend their employment than women, and this is largely explained by men having the above mentioned characteristics more often [35]. We have also compared health development among those extending their employment to those who retire early or at their pensionable age and found that voluntary extension of the working career beyond pensionable age does not have either positive or negative effects on health


For peer review only - http://bmjopen.bmj.com/site/about/guidelines.xhtml

and physical functioning among older workers [36]. Our results suggest that it would be important to develop and implement practices that support extended and flexible working life participation among older workers.

The complete list of publications can be found at the FIREA study website (https://sites.utu.fi/firea/en/publications/).

## Collaboration

Further information about the FIREA study can be found from the study website https://sites.utu.fi/firea/en/. The FIREA study welcomes research collaboration proposals that fall within the general aims of the project. We follow the EU general data protection regulation (679/2016) and the Finnish Data Protection Act (1050/2018) in data management and use. Anonymized partial datasets of the FIREA study are available by application with bona fide researchers with an established scientific record and bona fide organizations. For additional details, please contact principal investigator of the FIREA study Sari Stenholm.

## Data availability statement

Anonymized partial datasets of the FIREA study may be shared on a case-by-case basis upon request for bona fide researchers with an established scientific record and bona fide organizations. Data sharing outside the group is done in collaboration with FIREA group and requires a data-sharing agreement. Investigators can submit an expression of interest to FIREA research group at University of Turku, Finland (firea@utu.fi).

For peer review only

## Ethics approval

The FIREA study has been approved by the Ethics Committee of Hospital District of Southwest Finland (84/1801/2014). All FIREA participants have given written informed consent to participate in the study.

## Author contributions

S.S. and J.V. designed the cohort study; S.S. and K.S. drafted the manuscript; K.S. and J.P. performed the data analysis. All authors critically revised the manuscript and approved the final manuscript.

## Funding

The FIREA study has been financially supported by Academy of Finland [264944, 286294, 294154, 319246 and 332030]; Ministry of Education and Culture; Hospital District of Southwest Finland, Finnish State Grants for Clinical Research; Finnish Work Environment Fund [118060]; Juho Vainio Foundation; Signe and Ane Gyllenberg Foundation, Päivikki and Sakari Sohlberg Foundation, and The Finnish Foundation for Cardiovascular Research.

For peer review only

**Acknowledgements**

The FIREA study warmly thanks the study participants for their continuous commitment to the study. The research assistant Kati Pyykkö, study nurses Hanna Pynnönen and Minna Hyppönen, data manager Jesse Pasanen and other assisting personnel are acknowledged for their invaluable effort in the project.

**Conflict of interest**

None declared.

For peer review only

## References

1. Ekerdt DJ. Frontiers of research on work and retirement. *J Gerontol B Psychol Sci Soc Sci* 2010; **65B**(1): 69-80.

2. Behncke S. Does retirement trigger ill health? *Health Economics* 2012; **21**(3): 282-300.

3. Sjösten N, Kivimäki M, Singh-Manoux A, et al. Change in physical activity and weight in relation to retirement: the French GAZEL Cohort Study. *BMJ open* 2012; **2**: e000522-2011-. Print 2012.

4. Westerlund H, Kivimäki M, Singh-Manoux A, et al. Self-rated health before and after retirement in France (GAZEL): a cohort study. *Lancet* 2009; **374**(9705): 1889-96.

5. Westerlund H, Vahtera J, Ferrie JE, et al. Effect of retirement on major chronic conditions and fatigue: French GAZEL occupational cohort study. *BMJ* 2010; **341**: c6149.

6. Vahtera J, Westerlund H, Hall M, et al. Effect of retirement on sleep disturbances: the GAZEL prospective cohort study. *Sleep* 2009; **32**(11): 1459-66.

7. Barnett I, van Sluijs EM, Ogilvie D. Physical activity and transitioning to retirement: a systematic review. *American Journal of Preventive Medicine* 2012; **43**(3): 329-36.

8. Pulakka A, Leskinen T, Koster A, Pentti J, Vahtera J, Stenholm S. Daily physical activity patterns among aging workers: the Finnish Retirement and Aging Study (FIREA). *Occup Environ Med* 2019; **76**(1): 33-9.

9. Suorsa K, Pulakka A, Leskinen T, et al. Objectively Measured Sedentary Time Before and After Transition to Retirement: The Finnish Retirement and Aging Study. *The Journals of Gerontology: Series A* 2019; **75**(9): 1737-43.

10. Myllyntausta S, Pulakka A, Salo P, et al. Changes in accelerometer-measured sleep during the transition to retirement: the Finnish Retirement and Aging (FIREA) study. *Sleep* 2020; **43**(7): zsz318.

11. Suorsa K, Leskinen T, Pasanen J, et al. Changes in the 24-h movement behaviors during the transition to retirement: compositional data analysis. *Int J Behav Nutr Phys Act* 2022; **19**(1): 121.

12. Stenholm S, Pulakka A, Leskinen T, et al. Daily Physical Activity Patterns and Their Association With Health-Related Physical Fitness Among Aging Workers-The

For peer review only

Finnish Retirement and Aging Study. *J Gerontol A Biol Sci Med Sci* 2021; **76**(7): 1242-50.
13. Skotte J, Korshoj M, Kristiansen J, Hanisch C, Holtermann A. Detection of physical activity types using triaxial accelerometers. *J Phys Act Health* 2014; **11**(1): 76-84.
14. Stemland I, Ingebrigtsen J, Christiansen CS, et al. Validity of the Acti4 method for detection of physical activity types in free-living settings: comparison with video analysis. *Ergonomics* 2015; **58**(6): 953-65.
15. Suorsa K, Pulakka A, Leskinen T, et al. Comparison of Sedentary Time Between Thigh-Worn and Wrist-Worn Accelerometers. *Journal for the Measurement of Physical Behaviour* 2020; **3**(3): 234-43.
16. Brondeel R, Wasfi R, Perchoux C, et al. Is older adults' physical activity during transport compensated during other activities? Comparing 4 study cohorts using GPS and accelerometer data. *Journal of Transport & Health* 2019; **12**: 229-36.
17. Pasanen S, Halonen JI, Pulakka A, et al. Contexts of sedentary time and physical activity among ageing workers and recent retirees: cross-sectional GPS and accelerometer study. *BMJ Open* 2021; **11**(5): e042600.
18. Pasanen S, Halonen JI, Suorsa K, et al. Does work-related and commuting physical activity predict changes in physical activity and sedentary behavior during the transition to retirement? GPS and accelerometer study. *Health Place* 2023; **81**: 103025.
19. Finland S. Grid Database. 2023. https://www.stat.fi/tup/ruututietokanta/index_en.html.
20. Pulakka A, Leskinen T, Suorsa K, et al. Physical Activity across Retirement Transition by Occupation and Mode of Commute. *Med Sci Sports Exerc* 2020.
21. Suorsa K, Pulakka A, Leskinen T, Pentti J, Vahtera J, Stenholm S. Changes in prolonged sedentary behaviour across the transition to retirement. *Occupational and Environmental Medicine* 2021; **78**(6): 409-12.
22. Myllyntausta S, Salo P, Kronholm E, et al. Does removal of work stress explain improved sleep following retirement? The Finnish Retirement and Aging study. *Sleep* 2019; **42**(8): zsz109.
23. 2018 Physical Activity Guidelines Advisory Committee. 2018 Physical Activity Guidelines Advisory Committee Scientific Report: U.S. Department of Health and Human Services, 2018.

For Peer Review Only

24. Pahor M, Guralnik JM, Ambrosius WT, et al. Effect of Structured Physical Activity on Prevention of Major Mobility Disability in Older Adults: The LIFE Study Randomized Clinical Trial. *JAMA* 2014; **311**(23): 2387-96.

25. Singh B, Olds T, Curtis R, et al. Effectiveness of physical activity interventions for improving depression, anxiety and distress: an overview of systematic reviews. *British Journal of Sports Medicine* 2023: bjsports-2022-106195.

26. Lahdenperä M, Virtanen M, Myllyntausta S, Pentti J, Vahtera J, Stenholm S. Psychological Distress During the Retirement Transition and the Role of Psychosocial Working Conditions and Social Living Environment. *J Gerontol B Psychol Sci Soc Sci* 2022; **77**(1): 135-48.

27. KC P, Virtanen M, Törmälehto S, et al. Changes in life satisfaction during the transition to retirement: findings from the FIREA cohort study. *Eur J Ageing* 2022; **19**(4): 1587-99.

28. van der Heide I, van Rijn RM, Robroek SJ, Burdorf A, Proper KI. Is retirement good for your health? A systematic review of longitudinal studies. *BMC Public Health* 2013; **13**: 1180.

29. Syse A, Veenstra M, Furunes T, Mykletun RJ, Solem PE. Changes in Health and Health Behavior Associated With Retirement. *J Aging Health* 2017; **29**(1): 99-127.

30. Oshio T, Kan M. The dynamic impact of retirement on health: Evidence from a nationwide ten-year panel survey in Japan. *Prev Med* 2017; **100**: 287-93.

31. Stenholm S, Virtanen M, Pentti J, Oksanen T, Kivimaki M, Vahtera J. Trajectories of self-rated health before and after retirement: evidence from two cohort studies. *Occup Environ Med* 2020; **77**(2): 70-6.

32. KC P, Virtanen M, Kivimäki M, et al. Trajectories of work ability from mid-life to pensionable age and their association with retirement timing. *Journal of Epidemiology and Community Health* 2021; **75**(11): 1098-103.

33. Virtanen M, Myllyntausta S, Kauppi M, et al. Trajectories of Worktime Control From Midlife to Retirement and Working Beyond Retirement Age. *Work, Aging and Retirement* 2021; **8**(3): 273-81.

34. Kauppi M, Prakash KC, Virtanen M, et al. Social relationships as predictors of extended employment beyond the pensionable age: a cohort study. *Eur J Ageing* 2021; **18**(4): 491-501.

For peer review only

35. Myllyntausta S, Virtanen M, Pentti J, Kivimäki M, Vahtera J, Stenholm S. Why do men extend their employment beyond pensionable age more often than women? a cohort study. *European Journal of Ageing* 2022; **19**(3): 599-608.
36. KC P, Virtanen M, Pentti J, Kivimäki M, Vahtera J, Stenholm S. Does working beyond the statutory retirement age have an impact on health and functional capacity? The Finnish Retirement and Aging cohort study. *Occup Environ Med* 2020.

For peer review only

**Figure 1. Flow of the Finnish Retirement and Aging study (FIREA).**

**Figure 2. Schematic overview of the data collection in the Finnish Retirement and Aging study (FIREA).**

Bar color intensity indicates the working status of the participants. Light color = full time working, dark color = retired.

For peer review only

**Table 1. Baseline characteristics of the Finnish Retirement and Aging study (FIREA) participants and non-participants.**

| | Survey cohort (n=6,783) | Activity sub-study (n=908) | Clinical sub-study (n=290) | Non-participants (n=3,846) |
|---|---|---|---|---|
| Age (years), mean (SD) | 62.7 (1.2) | 62.4 (1.1) | 62.4 (1.0) | 62.7 (1.2) |
| Female, % | 82 | 85 | 83 | 75 |
| Occupational group, % | | | | |
| Manual | 40 | 33 | 31 | 45 |
| Lower non-manual | 30 | 29 | 34 | 28 |
| Upper non-manual | 30 | 38 | 35 | 26 |
| Married / cohabiting, % | 71 | 71 | 72 | |
| Physical activity (MET-hours per week) [a], mean (SD) | 23.6 (20.2) | 24.8 (20.0) | 28.8 (21.7) | |
| Body Mass Index (kg/m$^2$) [a], mean (SD) | 26.9 (4.6) | 26.6 (4.7) | 26.0 (4.0) | |
| Current smoking, % | 11 | 6 | 5 | |
| Alcohol risk use [b], % | 3 | 2 | 3 | |
| Good or rather good self-rated health [c], % | 73 | 77 | 82 | |


For peer review only - http://bmjopen.bmj.com/site/about/guidelines.xhtml

*Notes:* [a] Self-reported information; [b] Alcohol risk use: >24 units/week for men and >16 units/week for women. [c] Self-rated health was assessed by asking participants to rate their overall health status on a 5-point scale (1=good, 2=rather good, 3=average, 4=rather poor, 5=poor). MET = metabolic equivalent.

For peer review only

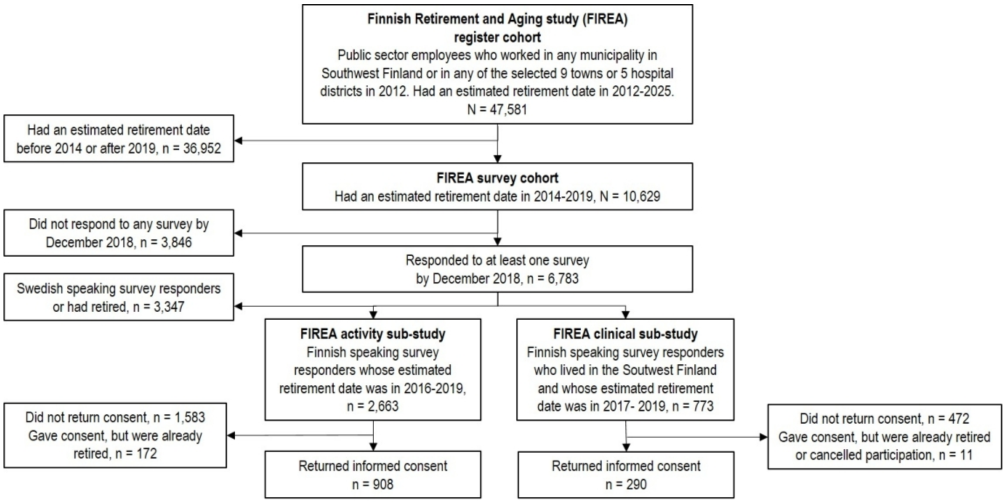


Figure 1. Flow of the Finnish Retirement and Aging study (FIREA).

257x128mm (150 x 150 DPI)

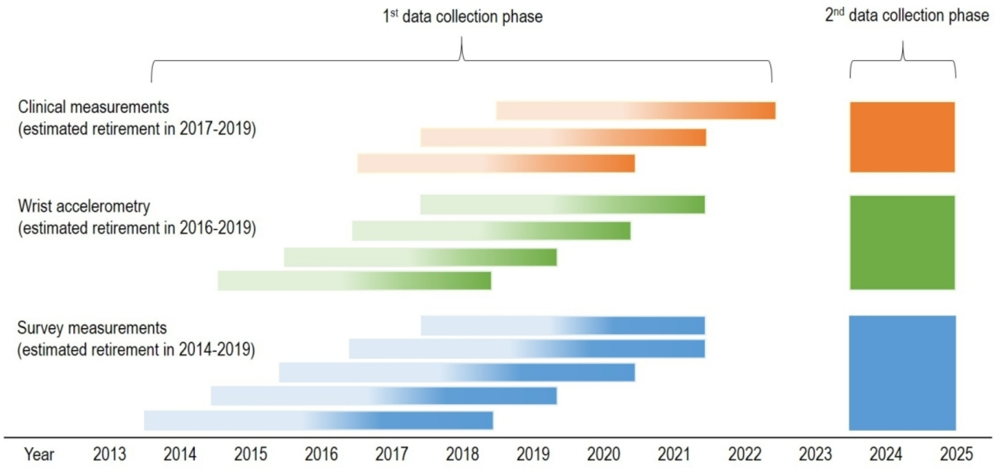


Figure 2. Schematic overview of the data collection in the Finnish Retirement and Aging study (FIREA).

Bar color intensity indicates the working status of the participants. Light color = full time working, dark color = retired.

270x133mm (150 x 150 DPI)

**Supplementary Table 1. Overview of the data collection methods in the Finnish Retirement and Aging study.**

| | |
|---|---|
| **Survey questionnaire** | |
| Sociodemographic characteristics | Gender, marital status |
| Health and wellbeing | Self-rated health, chronic conditions, vision, hearing, pain [1], height, weight, physical functioning (Short Form Health Survey (SF-36)) [1], falls, subjective memory complaints, depression [2,3], psychological wellbeing (General Health Questionnaire (GHQ)-12) [4], optimism and pessimism (Life Orientation test) [5], life satisfaction [6], sexual health (Female Sexual Function Index)[7], relationship happiness |
| Health behaviors | Sleep duration [8], sleep difficulties (Jenkins Sleep Problem Scale) [9], daytime tiredness [8], napping, sitting [10], leisure and commuting physical activity [11], smoking, alcohol consumption, diet |
| Social and living environment | Social networks [12], social participation [13], family caregiving, negative life events, neighborhood cohesion [14], health care use |
| Work and retirement | Workability [15], worktime mode, psychosocial stressors (job strain, work demands and control [16], worktime control [17], effort-reward imbalance [18], organizational justice [19]), physical work stressors (strenuous work, awkward working positions, repetitive work), plans for retirement timing, actual retirement date |
| | |
| **Device-based movement behavior measures** | |
| ActiGraph accelerometer (wActiSleep-BT and wGT3X-BT), wrist-worn | Total amount of physical activity as vector magnitude counts per minute (VM CPM) [20]. Time spent sedentary, in light physical activity, in moderate-to-vigorous physical activity, bouts (number and duration) of sedentary time, light physical activity and moderate-to-vigorous physical activity [21,22], sleep duration [22,23], sleep efficiency [23], bedtime and awakening time, ambient light [24] |

For peer review only

| | |
|---|---|
| Axivity accelerometer (AX3), thigh-worn | Time spent lying, sitting, standing, moving, walking slow, walking fast, stair walking, running, cycling and other physical activity. Number of steps and sit-to-stand transitions. Duration of lying, sitting and standing bouts [25,26] |
| Global Positioning System and accelerometer device (SenseDoc V.2.0), waist-worn | Time spent sedentary, in light physical activity and in moderate-to-vigorous physical activity in different locations and during travel [27-29]. |
| | |
| **Clinical measurements** | |
| Anthropometry and body composition | Weight, height, body mass index, waist circumference, body composition (Inbody 720, Biospace Co., Seoul, Korea) [30]. |
| Cardiovascular measurements | Office blood pressure, 24-h ambulatory blood pressure [31], carotid-femoral Pulse Wave Velocity [32], ankle brachial index. |
| Physical fitness | VO2peak measured with indirect submaximal bicycle ergometer test [30], modified push-up test [33], sit-up test [33]. |
| Physical functioning | Maximal and normal walking speed over 4 meters [34], Short Physical Performance Battery [35], 1-leg standing balance [36], hand grip strength (Jamar dynamometer) . |
| Cognitive functioning | Cambridge Neuropsychological Test Automated Battery (CANTAB®), computerized test battery covering multiple cognitive domains including learning and memory, working memory, information processing, and reaction time [37], Trail Making Test [38], Mini Mental State Examination and verbal recall from the Consortium to Establish a Registry for Alzheimer's Disease (CERAD) [39]. |
| Blood samples | Fasting blood samples taken, fasting glucose, lipids, insulin, C-reactive protein assayed. Samples stored at –80°C. |
| Hair sample | Cortisol, cortisone. |
| Self-reported medication use | ATC-codes |
| | |

For peer review only

| Register data | |
|---|---|
| Sociodemographic characteristics | Date of birth, sex, occupational code, employment history (Pension insurance institute Keva) |
| National health registers | Primary diagnosis and date of hospital discharge from years 1987-2019 (Care Register for Health Care maintained by the Finnish Institute for Health and Welfare); medication purchase, special reimbursement, rehabilitation, sick-leave days from years 1994-2019 (Registers of the Social Insurance Institution of Finland); type of cancer, date of diagnosis years 1953-2019 (Cancer register) |
| Occupational injuries | Types and causes of workplace and commuting injuries from years 2000-2019 (the Federation of Accident Insurance Institutions) |
| Residential environment characteristics | Home address; socioeconomic disadvantage: median household income, low educational attainment (percentage of people over 18 years old with low education) and unemployment rate from each 250x250m square [40]; Greenness in the neighbourhood calculated as Normalised Difference Vegetation Index (NDVI) for each map grip from a satellite image composite using Google Earth Engine [40]. Mean, maximum and minimum temperature and humidity data from >500 weather stations over Finland interpolated to a spatial resolution of 1x1km$^2$ to measure continuous daily gridded time series of daily temperature. |

For peer review only

## References


1. Hayes R, Sherbourne C, Mazel R. The RAND 36-item Health Survey 1.0. *Health Economics* 1993; **2**: 217–27.
2. Arroll B, Khin N, Kerse N. Screening for depression in primary care with two verbally asked questions: cross sectional study. *Bmj* 2003; **327**(7424): 1144-6.
3. Beck AT, Ward CH, Mendelson M, Mock J, Erbaugh J. An inventory for measuring depression. *Arch Gen Psychiatry* 1961; **4**: 561-71.
4. Goldberg DP. The detection of psychiatric illness by questionnaire. Oxford: Oxford university press; 1972.
5. Kronström K, Karlsson H, Nabi H, et al. Optimism and pessimism as predictors of work disability with a diagnosis of depression: a prospective cohort study of onset and recovery. *J Affect Disord* 2011; **130**(1-2): 294-9.
6. Koivumaa-Honkanen H, Honkanen R, Viinamäki H, Heikkilä K, Kaprio J, Koskenvuo M. Self-reported life satisfaction and 20-year mortality in healthy Finnish adults. *Am J Epidemiol* 2000; **152**(10): 983-91.
7. Rosen R, Brown C, Heiman J, et al. The Female Sexual Function Index (FSFI): a multidimensional self-report instrument for the assessment of female sexual function. *Journal of sex & marital therapy* 2000; **26**(2): 191-208.
8. Myllyntausta S, Salo P, Kronholm E, et al. Does removal of work stress explain improved sleep following retirement? The Finnish Retirement and Aging study. *Sleep* 2019; **42**(8): zsz109.
9. Jenkins CD, Stanton BA, Niemcryk SJ, Rose RM. A scale for the estimation of sleep problems in clinical research. *Journal of clinical epidemiology* 1988; **41**(4): 313-21.
10. Leskinen T, Pulakka A, Heinonen OJ, et al. Changes in non-occupational sedentary behaviours across the retirement transition: the Finnish Retirement and Aging (FIREA) study. *J Epidemiol Community Health* 2018; **72**(8): 695-701.
11. Kujala UM, Kaprio J, Sarna S, Koskenvuo M. Relationship of leisure-time physical activity and mortality: the Finnish twin cohort. *Journal of American Medical Association* 1998; **279**(6): 440-4.
12. Antonucci T. Measuring social support networks: Hierarchical mapping technique. *Generations: Journal of the American Society on Aging* 1986; **10**(4): 10-2.
13. Kauppi M, Prakash KC, Virtanen M, et al. Social relationships as predictors of extended employment beyond the pensionable age: a cohort study. *Eur J Ageing* 2021; **18**(4): 491-501.


For peer review only

14. Lahdenperä M, Virtanen M, Myllyntausta S, Pentti J, Vahtera J, Stenholm S. Psychological Distress During the Retirement Transition and the Role of Psychosocial Working Conditions and Social Living Environment. *J Gerontol B Psychol Sci Soc Sci* 2022; **77**(1): 135-48.
15. Ilmarinen J, Tuomi K, Klockars M. Changes in the work ability of active employees over an 11-year period. *Scand J Work Environ Health* 1997; **23 Suppl 1**: 49-57.
16. Karasek R, Brisson C, Kawakami N, Houtman I, Bongers P, Amick B. The Job Content Questionnaire (JCQ): an instrument for internationally comparative assessments of psychosocial job characteristics. *J Occup Health Psychol* 1998; **3**(4): 322-55.
17. Ala-Mursula L, Vahtera J, Linna A, Pentti J, Kivimäki M. Employee worktime control moderates the effects of job strain and effort-reward imbalance on sickness absence: the 10-town study. *J Epidemiol Community Health* 2005; **59**(10): 851-7.
18. Siegrist J, Wege N, Pühlhofer F, Wahrendorf M. A short generic measure of work stress in the era of globalization: effort-reward imbalance. *International archives of occupational and environmental health* 2009; **82**(8): 1005-13.
19. Elovainio M, Kivimäki M, Vahtera J. Organizational justice: evidence of a new psychosocial predictor of health. *Am J Public Health* 2002; **92**(1): 105-8.
20. Pulakka A, Leskinen T, Koster A, Pentti J, Vahtera J, Stenholm S. Daily physical activity patterns among aging workers: the Finnish Retirement and Aging Study (FIREA). *Occup Environ Med* 2019; **76**(1): 33-9.
21. Suorsa K, Leskinen T, Pasanen J, et al. Changes in the 24-h movement behaviors during the transition to retirement: compositional data analysis. *Int J Behav Nutr Phys Act* 2022; **19**(1): 121.
22. Migueles JH, Rowlands AV, Huber F, Sabia S, van Hees VT. GGIR: A research Community–Driven open source R package for generating physical activity and sleep outcomes from multi-day raw accelerometer data. *Journal for the Measurement of Physical Behaviour* 2019; **2**(3): 188-96.
23. Myllyntausta S, Pulakka A, Salo P, et al. Changes in accelerometer-measured sleep during the transition to retirement: the Finnish Retirement and Aging (FIREA) study. *Sleep* 2020; **43**(7): zsz318.
24. Flynn JI, Coe DP, Larsen CA, Rider BC, Conger SA, Bassett DR, Jr. Detecting indoor and outdoor environments using the ActiGraph GT3X+ light sensor in children. *Med Sci Sports Exerc* 2014; **46**(1): 201-6.
25. Skotte J, Korshoj M, Kristiansen J, Hanisch C, Holtermann A. Detection of physical activity types using triaxial accelerometers. *J Phys Act Health* 2014; **11**(1): 76-84.
26. Stemland I, Ingebrigtsen J, Christiansen CS, et al. Validity of the Acti4 method for detection of physical activity types in free-living settings: comparison with video analysis. *Ergonomics* 2015; **58**(6): 953-65.

For peer review only

27. Thierry B, Chaix B, Kestens Y. Detecting activity locations from raw GPS data: a novel kernel-based algorithm. *International journal of health geographics* 2013; **12**: 14.
28. Pasanen S, Halonen JI, Pulakka A, et al. Contexts of sedentary time and physical activity among ageing workers and recent retirees: cross-sectional GPS and accelerometer study. *BMJ Open* 2021; **11**(5): e042600.
29. Pasanen S, Halonen JI, Suorsa K, et al. Does work-related and commuting physical activity predict changes in physical activity and sedentary behavior during the transition to retirement? GPS and accelerometer study. *Health Place* 2023; **81**: 103025.
30. Stenholm S, Pulakka A, Leskinen T, et al. Daily Physical Activity Patterns and Their Association With Health-Related Physical Fitness Among Aging Workers-The Finnish Retirement and Aging Study. *J Gerontol A Biol Sci Med Sci* 2021; **76**(7): 1242-50.
31. Karelius S, Vahtera J, Suorsa K, et al. Changes in ambulatory blood pressure during the transition to retirement. *J Hypertens* 2023; **41**(1): 187-93.
32. Lindroos AS, Johansson JK, Puukka PJ, et al. The association between home vs. ambulatory night-time blood pressure and end-organ damage in the general population. *J Hypertens* 2016; **34**(9): 1730-7.
33. Suorsa K, Mattila VM, Leskinen T, et al. Work ability and physical fitness among aging workers: the Finnish Retirement and Aging Study. *Eur J Ageing* 2022; **19**(4): 1301-10.
34. Guralnik JM, Ferrucci L, Simonsick EM, Salive ME, Wallace RB. Lower-extremity function in persons over the age of 70 years as a predictor of subsequent disability. *New England Journal of Medicine* 1995; **332**: 556-61.
35. Guralnik JM, Simonsick EM, Ferrucci L, et al. A Short Physical Performance Battery Assessing Lower Extremity Function: Association With Self-Reported Disability and Prediction of Mortality and Nursing Home Admission. *J Gerontol* 1994; **49**(2): M85-M94.
36. Suni JH, Oja P, Miilunpalo SI, Pasanen ME, Vuori IM, Bos K. Health-related fitness test battery for adults: associations with perceived health, mobility, and back function and symptoms. *Arch Phys Med Rehabil* 1998; **79**(5): 559-69.
37. Teräs T, Rovio S, Spira AP, et al. Associations of accelerometer-based sleep duration and self-reported sleep difficulties with cognitive function in late mid-life: the Finnish Retirement and Aging Study. *Sleep Med* 2020; **68**: 42-9.
38. Reitan RM. Trail Making Test: Manual for Administration and Scoring. Tuscon, AZ: Reitan Neuropsychology Laboratory, 1992.
39. Morris JC, Heyman A, Mohs RC, et al. The consortium to establish a registry for Alzheimer´s disease (CERAD). Part 1. Clinical and neuropsychological assessement of Alzheimer´s disease. *Neurology* 1989; **39**: 1159-65.

For peer review only

40. Halonen JI, Pulakka A, Pentti J, et al. Cross-sectional associations of neighbourhood socioeconomic disadvantage and greenness with accelerometer-measured leisure-time physical activity in a cohort of ageing workers. *BMJ Open* 2020; **10**(8): e038673.

For peer review only

BMJ Open

# Cohort Profile: Finnish Retirement and Aging study (FIREA), a prospective cohort study

| | |
|---|---|
| Journal: | *BMJ Open* |
| Manuscript ID | bmjopen-2023-076976.R1 |
| Article Type: | Cohort profile |
| Date Submitted by the Author: | 20-Oct-2023 |
| Complete List of Authors: | Stenholm, Sari; University of Turku, Department of Public Health; Turku University Hospital<br>Suorsa, Kristin; University of Turku, Department of Public Health<br>Leskinen, Tuija; Turun Yliopisto<br>Myllyntausta, Saana; Turun Yliopisto, Department of Psychology and Speech-Language Pathology<br>Pulakka, Anna; University of Oulu; Finnish Institute for Health and Welfare<br>Pentti, Jaana; University of Turku, Department of Public Health<br>Vahtera, Jussi; Turun Yliopisto, Dep Public Health; Finnish Institute of Occupational Health, |
| <b>Primary Subject Heading</b>: | Public health |
| Secondary Subject Heading: | Epidemiology |
| Keywords: | Aging, OCCUPATIONAL & INDUSTRIAL MEDICINE, PREVENTIVE MEDICINE, PUBLIC HEALTH, EPIDEMIOLOGIC STUDIES |
| | |

SCHOLARONE™
Manuscripts

*I, the Submitting Author has the right to grant and does grant on behalf of all authors of the Work (as defined in the below author licence), an exclusive licence and/or a non-exclusive licence for contributions from authors who are: i) UK Crown employees; ii) where BMJ has agreed a CC-BY licence shall apply, and/or iii) in accordance with the terms applicable for US Federal Government officers or employees acting as part of their official duties; on a worldwide, perpetual, irrevocable, royalty-free basis to BMJ Publishing Group Ltd ("BMJ") its licensees and where the relevant Journal is co-owned by BMJ to the co-owners of the Journal, to publish the Work in this journal and any other BMJ products and to exploit all rights, as set out in our licence.*

*The Submitting Author accepts and understands that any supply made under these terms is made by BMJ to the Submitting Author unless you are acting as an employee on behalf of your employer or a postgraduate student of an affiliated institution which is paying any applicable article publishing charge ("APC") for Open Access articles. Where the Submitting Author wishes to make the Work available on an Open Access basis (and intends to pay the relevant APC), the terms of reuse of such Open Access shall be governed by a Creative Commons licence – details of these licences and which Creative Commons licence will apply to this Work are set out in our licence referred to above.*

*Other than as permitted in any relevant BMJ Author's Self Archiving Policies, I confirm this Work has not been accepted for publication elsewhere, is not being considered for publication elsewhere and does not duplicate material already published. I confirm all authors consent to publication of this Work and authorise the granting of this licence.*

For peer review only

# Cohort Profile: Finnish Retirement and Aging study (FIREA), a prospective cohort study

Sari Stenholm[1,2,3], Kristin Suorsa[1,2], Tuija Leskinen[1,2], Saana Myllyntausta[4], Anna Pulakka[5,6], Jaana Pentti[1,2,7], Jussi Vahtera[1,2]

[1] Unit of Public Health, University of Turku and Turku University Hospital, Turku, Finland;

[2] Centre for Population Health Research, University of Turku and Turku University Hospital; Turku, Finland;

[3] Research Services, Turku University Hospital and University of Turku, Finland

[4] Department of Psychology and Speech-Language Pathology, University of Turku, Turku, Finland

[5] Research Unit of Population Health, Faculty of Medicine, University of Oulu, Oulu, Finland

[6] Population Health Unit, Finnish Institute for Health and Welfare, Helsinki, Finland

[7] Clinicum, Faculty of Medicine, University of Helsinki, Helsinki, Finland

Correspondence:

Prof. Sari Stenholm, Department of Public Health, University of Turku, Finland

Postal address: Public Health, University of Turku, FI-20014 University of Turku, Finland

Email: sari.stenholm@utu.fi ; Telephone: +358 50 465 1745

For peer review only

## ABSTRACT

**Purpose:** The Finnish Retirement and Aging study (FIREA) was set up to study changes in health behavioral and cardiometabolic risk factors across retirement transition, and to examine the long-term consequences of work and retirement on health and functioning with advancing age.

**Participants:** Public sector workers whose estimated statutory retirement date was in 2014–2019 were invited to participate by sending them a questionnaire 18 months prior to their estimated retirement date. In the first phase of the FIREA study, participants were followed up with annual surveys, accelerometer and clinical measurements during retirement transition into post-retirement years. The FIREA survey cohort includes 6,783 participants, of which 908 belong also to the activity sub-study, and 290 to the clinical sub-study.

**Findings to date:** Collected data includes survey measures about health, lifestyle factors, psychosocial distress, work-related factors as well as retirement intentions. Accelerometer and GPS devices are used to measure 24-hour movement behaviors. Clinical examination includes blood and hair sample, measurements of anthropometry, cardiovascular function, physical fitness, physical and cognitive function. Our results suggest that in general retirement transition seems to have beneficial influence on health behaviors as well as on physical and mental health, but there are large individual differences, and certain behaviors such as sedentariness tend to increase especially among those retiring from manual occupations.

**Future plans:** The second phase of the FIREA study will be conducted during 2023–2025, when participants are 70 years old. The FIREA study welcomes research collaboration proposals that fall within the general aims of the project.

For peer review only

**Key words:** Aging, preventive medicine, public health, retirement, cohort study

For peer review only



For peer review only - http://bmjopen.bmj.com/site/about/guidelines.xhtml

## Strengths and limitations of this study

- The unique feature of the FIREA study is that the data collection was targeted around estimated statutory retirement date and it continued annually from final working years into actual retirement.
- In addition to survey measures, which are prone to response bias, participants have been followed with accelerometer and clinical measurements, which are more accurate and enable capturing subtle changes in movement behaviors and cardiometabolic risk markers.
- The extensive Finnish national registers offer a possibility to complement collected data with information on participants' health, use of health care services as well as on neighborhood characteristics.
- The participation rate in the first phase of the FIREA study has been good, especially among those who participated in the activity (≥88% across measurements years) and the clinical sub-study (≥92% across measurements years).
- The main limitation of the FIREA study is a healthy worker effect as the inclusion criteria include participation in the working life after the age of 60 (those retired due to illness, disability or unemployed are not included in the study population); moreover, men and individuals living alone are under-represented, which further limits the generalizability of the results.

For peer review only - http://bmjopen.bmj.com/site/about/guidelines.xhtml

## Introduction

The Finnish Retirement and Aging study (FIREA) was established in 2013 to understand in detail the short and long term effects of work and retirement, an important life transition in late midlife, on people's health behaviors and health. Specifically, the FIREA study was initiated 1) to examine changes in health behaviors, cardiometabolic risk factors, and health indicators during retirement transition by following older workers from final working years to full-time retirement; 2) to study individual- and work-related factors that predict working life participation in people aged 60 years and older; and 3) to examine long-term consequences of work and retirement on health and functioning with an advancing age.

Transitioning from working life into retirement brings flexibility to daily time use, while work-related stressors are also removed, which may have extensive impact on health behaviors and social activities and consequently on health and well-being. At the time FIREA study was initiated, several studies on retirement and health had been published (1-4), and there were several large cohort studies following up participants from age 50 onwards, such as the Health and Retirement Study (HRS) in the US (5), the English Longitudinal Study on Aging (ELSA) in the UK (6) and the Survey of Health, Ageing and Retirement in Europe (SHARE) from several European countries (7). However, only a few studies had studied changes in health behaviors by following individuals through retirement transition. These studies had reported changes in health behaviors, such as increased physical activity and improved sleep (8-12), but the results were contrasting, and information on health and health behaviors was based on self-reported measures. To identify subtle changes in health behaviors and clinical risk markers, and to overcome measurement bias related to survey measures, the FIREA study took one step further by establishing annual device-based and clinical measurements for older

For peer review only

workers close to their estimated retirement data. These measurements have been complemented with annual surveys, including questions about lifestyle factors, physical and mental health, work and social factors, as well as with register-based information on participants' living environment and linkage to national health registries. The aim is to continue following up the study participants until old age.

## Cohort description

### *Study population and data collection*

The eligible study population of the FIREA cohort comprised of public sector employees who were working in any town in Southwest Finland or in any of the a priori selected 10 towns or five hospital districts around Finland in 2012 and whose estimated statutory retirement date was between 2012 and 2025 (N=47,581). This register cohort was acquired from the pension insurance institute for the municipal sector in Finland (Keva), and included information about workers' date of birth, work history and estimated retirement date. Individuals whose estimated retirement date was between 2014 and 2019, were invited to participate in the FIREA study by sending them a questionnaire 18 months prior to their estimated retirement date (N=10,629). The questionnaire included information sheet and informed consent. The data collection started in the fall 2013. In the first phase of the FIREA study, questionnaires were sent once a year with an aim to gather data from at least two time points before and two time points after the transition to statutory retirement. In total, 6,783 participants had responded to at least one questionnaire by the end of 2018. 5,506 participants responded at least in two time points and of them 3,948 provided response before and after retirement. Flow chart of the FIREA study is shown in Figure 1, and schematic overview of the data collection in Figure 2.

For peer review only

In 2014, a FIREA activity sub-study was launched and Finnish-speaking FIREA survey cohort participants who were still working and had an estimated statutory retirement date between 2016 and 2019 were invited to participate by mailing them a separate information sheet and informed consent (n=2,663). Of them, 908 consented to participate and they were sent the accelerometer on average 2.1 months (SD 1.2) after completing the questionnaire. They were then followed via accelerometer measurements and survey questionnaires annually, at the same time of the year, up to five times in total.

One year later in 2015, a FIREA clinical sub-study was initiated. Finnish-speaking survey cohort participants who were still working, had an estimated statutory retirement date between 2017 and 2019 and who lived in the Southwest Finland were invited to participate by mailing them a separate information sheet and informed consent (n=773). Of them, 290 consented to participate in annual clinical examination and the first visit took place on average 4.3 months (SD 3.4) after completing the questionnaire. Up to five annual follow-up visits were conducted until the participants had retired. Participants did not receive any reimbursement for taking part in the study.

Due to COVID-19 pandemic and consequent national restrictions, the data collection for activity and clinical sub-study were temporarily discontinued between March and June in 2020. However, this influenced only a small proportion of the participants, since the majority of the participants had already retired and completed the first phase of the data collection before 2020.

Table 1 provides comparison between the participants in the survey cohort, the activity sub-study, the clinical sub-study, and the non-participants. In comparison to the FIREA participants, the proportions of men and manual workers were greater among the non-participants. Participants in the survey cohort, the activity sub-study,



For peer review only - http://bmjopen.bmj.com/site/about/guidelines.xhtml

For peer review only

and the clinical sub-study did not differ from each other in terms of age, sex, and marital status, but the proportions of manual workers and current smokers were greater in the survey cohort compared to activity sub-study and clinical sub-study participants. Self-reported physical activity was highest and self-rated health better among the clinical sub-study participants and lowest and less optimal, respectively, in the survey cohort.

In the first phase of the FIREA study (2013–2022), the data collection took place annually, always at the same time of the year. On average, the participants provided survey data on 3.6 measurement points (range 1–7, SD 1.4). The mean number of annual measurements in the activity sub-study was 3.7 (range 1–5, SD 0.7) and in the clinical sub-study 3.0 (range 1–5, SD 0.6).

Of the initial survey cohort, 58% have both pre- and post-retirement data (n=3,948), because many of the initial cohort had already retired when they were contacted for the first time. The actual retirement date was obtained from the survey and this information was used to identify pre- and post-retirement measurements. Respective proportions for the pre- and post-retirement measurements are 83% for the activity sub-study and 88% for the clinical sub-study. The higher proportions compared to the survey cohort reflect the fact that only those who were still working were invited to participate in these sub-studies.

*Retirement system in Finland*

In Finland, from 2005 onwards, public sector employees have been able to retire on a statutory basis after the age of 63 years, but at the latest before the age of 68 years. Following a pension reform in 2017, each age group has their own retirement age, which is tied to the life expectancy. Postponing retirement from the estimated

For peer review only

retirement age will accrue pension income level. After the pension reform, employees may also retire on partial early old-age pension, years of-service pension (for those who have worked for at least 38 years in job that requires great mental or physical effort), or on full or partial disability pension (13). The FIREA study participants were, due to the inclusion criteria, mostly retiring due to old age with a mean age of 64.0 years (SD 1.4).

*Measurements*

*Survey measurements.* In the first phase of the FIREA study, annual surveys included questions about health, chronic diseases, physical functioning, memory, lifestyle factors, psychosocial distress, social networks, work-related physical and psychological stressors as well as retirement intentions and actual retirement date. The questionnaire was 20 pages long and it took about 20-30 minutes to complete. The content of the questionnaire was similar in each follow-up year. Details of the survey instruments are provided in Supplement Table 1. In addition to the multiple-choice questions, the survey included open-end questions regarding positive expectations and fears about retirement for those who were still working, and about positive and negative consequences of retirement for those who had retired.

*Activity sub-study measurements.* Participants' 24-hour movement behaviors were measured using wrist-worn triaxial ActiGraph wActiSleep-BT and wGT3X-BT accelerometers (ActiGraph, Pensacola, Florida, US), which were mailed to the participants. Participants were asked to wear the accelerometer on their non-dominant wrist for seven consecutive days and six consecutive nights at all times and fill in a diary of their workdays and days off, daily bedtimes and modes of commute. Details of the FIREA accelerometer measurement protocol has been



For peer review only - http://bmjopen.bmj.com/site/about/guidelines.xhtml

described earlier (14). Moreover, accelerometer data reduction procedures have been described in detail earlier (14-17).

*Clinical sub-study measurements.* Annual clinical examination visits were conducted at the University of Turku, in the Faculty of Medicine's research facilities. Measurements were conducted in the morning by trained study nurses and the visit lasted about 2.5–3 hours. Participants received feedback of the measurements for which results were available.

A 10-hour fasting blood sample were drawn by venipuncture in the morning shortly after arriving to the clinic. All blood samples were aliquoted at the same day and then stored at -80°C. Plasma cholesterol (total, low-density lipoprotein cholesterol, high-density lipoprotein cholesterol), triglycerides, fasting insulin and glucose and C-reactive protein were analyzed later at the laboratory of Turku University Hospital, Finland.

For the hair sample a strand of hair was cut from a standardized area of the posterior vertex region of the head as close to the scalp as possible (18) Hair samples were stored in foil in a dry place protected from light. Hair cortisol and cortisone were analyzed later using mass spectrometry (Technical University of Dresden, Germany).

For anthropometrics, weight, height, body mass index and waist circumference were measured by using standard methods. Body composition was measured with Inbody 720 (Biospace Co., Seoul, Korea) (19). Systolic and diastolic blood pressure was measured from both arms and ankles (Microlife WatchBP Office Central, Microlife AG, Widnau, Switzerland) (20), providing information also on ankle brachial index.

Physical function measures included maximal and normal walking speed over 4 meters (21), Short Physical Performance Battery (walking speed, balance and chair

For peer review only

rise test) (22) and 1-leg standing balance (23). In addition, maximal handgrip strength was measured on the dominant hand in a sitting position with Jamar dynamometer following protocol used in earlier large-scale aging studies (24).

Cognitive testing included Cambridge Neuropsychological Test Automated Battery (CANTAB®), computerized test battery covering multiple cognitive domains including learning and memory, working memory, information processing, and reaction time (25). In addition, Trail Making Test (26), Mini Mental State Examination and verbal recall from the Consortium to Establish a Registry for Alzheimer's Disease (CERAD) (27).

During the clinical visit, the participants also self-completed depression (Beck's depression inventory) (28) and dietary questionnaires, which included questions about eating rhythm and indicators of healthy diet. In addition, participants were given a sexual health questionnaire to be filled at home, which was then returned back to the university. Details of these measurements are provided in Supplement Table 1.

Prior to the clinical examination, the participants received a wrist-worn triaxial ActiGraph wActiSleep-BT accelerometer via mail and were instructed to wear it continuously on their non-dominant wrist for 24 hours per day for at least seven days and nights, including at least two workdays and two days off. Detailed description about the wrist-worn accelerometer data collection and data reduction procedures applied to the clinical sub-study have been provided elsewhere (19).

During the clinical examination visit, the participants were provided two additional devices to measure their movement behavior and the study nurse instructed the use of these devices. While using the devices the participants were asked to fill in a daily diary, in which they provided information on their workdays and days off, and on their daily bedtimes and modes of commute. The study nurse

For peer review only

fastened a triaxial Axivity AX3 accelerometer (Axivity Ltd., Newcastle, UK) to the participants' right thigh using adhesive waterproof film dressing. Participants were asked to wear the accelerometer for at least two workdays and two days off at all times when still working, and a minimum of any four days when retired. Fastening the accelerometer to thigh enables identification of postures such as sitting and standing and types of physical activity such as walking, running, and cycling based on thigh angle and acceleration (29, 30). Detailed measurement protocol and accelerometer data reduction procedures for thigh-worn accelerometers have been described elsewhere (31). In addition, participants received a waist-worn SenseDoc 2.0 device (Mobysens Technologies Inc, Canada), which includes a Global Positioning System (GPS) sensor and a tri-axial accelerometer (32). The combined GPS and accelerometer data provide information about intensity of the physical activity, but also about activity contexts and locations that is home, transportation, and allows linking environmental characteristics to these locations. Participants were requested to wear the device during waking hours for at least two workdays and two days off when still working, and a minimum of any four days when retired. Details of the measurements and data processing have been described earlier (33, 34). Participants were also requested to undergo a 24-hour ambulatory blood pressure measurement after the clinical visit. (20) The measurement was performed with a Microlife WatchBP O3 Monitor (Microlife AG, Widnau, Switzerland). Blood pressure was measured every 30 minutes over the entire 24 hours. Participants reported times of going to bed and waking up, which were used to calculate average awake and asleep blood pressure.

Within one month from the initial clinical examination visit, physical fitness tests (n=270) were performed by an experienced exercise physiologist at the Paavo Nurmi Centre, Sports & Exercise Medicine Unit in connection with University of Turku and



For peer review only - http://bmjopen.bmj.com/site/about/guidelines.xhtml

pulse wave velocity measurement (n=260) was conducted by study nurse at the university research facilities. Physical fitness testing included individually performed muscular fitness tests, modified push-up test and sit-up test, which are described in detail elsewhere (19), (35). In addition, cardiorespiratory fitness was measured in a group of 3–4 persons with indirect submaximal bicycle ergometer test according to the American College of Sports Medicine guidelines (19, 36).

Arterial stiffness was assessed as carotid-femoral pulse wave velocity measurement (SphygmoCor PVx with MM3 electronic module and Millar tonometer), which are described in detail elsewhere (37). Details of the clinical measurements are provided in Supplement Table 1.

*Linkage to register data.* Data collected from the study participants have been complemented with national register data and the linkage is conducted by using the national personal identification numbers (unique number assigned to all Finnish residents). Health-related outcomes are available until the end of 2019 from the hospital discharge information recorded by the Finnish Institute for Health and Welfare and the Cancer Register. In addition, medication purchase, special reimbursement, rehabilitation, sick-leave days are available from the registers of the Social Insurance Institution of Finland, and types and causes of workplace and commuting injuries from the Federation of Accident Insurance Institutions. Details of the register data are provided in Supplement Table 1.

Several indicators of the neighbourhood characteristics, such as a standardized index for socioeconomic disadvantage, degree of greenness, and daily temperature in the neighbourhood, have been derived by using information on participants' geocoded residential addresses from the Population Register Centre of Finland and these data were positioned to the Statistics Finland Grid Database, a


For peer review only - http://bmjopen.bmj.com/site/about/guidelines.xhtml

250×250m map grid that covers the whole country (38). Details of these neighborhood-level indicators are provided in Supplement Table 1.

*Patient and public involvement*

Study population or the general public were not involved in the planning, design or conduction of the study.

## Findings to date

*Movement behaviors*

One of the focuses of the FIREA study has been to understand how movement behaviors change when people transition from full time work to retirement. By using annually collected wrist-accelerometer data and by addressing differences between sex and occupations, we have shown that women retiring from manual occupations decrease their physically activity, whereas men from non-manual occupations slightly increase physical activity after retirement (39). Despite the declining trend among women, they are still more active than men both before and after retirement. Our results also suggest that sleep duration and sedentary time, especially among women retiring from manual occupations, increase after retirement (15, 16, 40, 41). More recently, we have applied compositional data analysis (CoDA), which allows examination of concomitant changes of the 24-h movement behaviors, that is physical activity, sedentary time, and sleep (17). Our findings suggest that retirement decreases the proportion of time spent in active behaviors, especially moderate-to-vigorous physical activity, in relative to time spent in passive behaviors, such as sedentary time and sleep. These findings highlight that retirement is an important time period in late midlife for paying special attention to activity behavior and develop new daily routines, which can replace work- and commuting-related activities and



For peer review only - http://bmjopen.bmj.com/site/about/guidelines.xhtml

involve physical activity in different forms. With advancing age, the importance of physically active lifestyle is emphasized, because it helps not only to prevent and treat non-communicable diseases, such as cardiovascular disease, type 2 diabetes, and various cancers (42), but it also helps to maintain physical functioning (43) and improve mental well-being (44).

*Health and mental well-being*

In addition to movement behaviors, we have also been interested in retirement-induced changes in various health and wellbeing indicators. We have shown that psychological distress decreases drastically after retirement and especially among those with poorer psychosocial working conditions and social living environment (45). Similarly, life satisfaction increases, most prominently among women, those with suboptimal health, and those living without a spouse (46). Previous studies have reported both improvement and decline in self-reported health after retirement (9, 47-49), which was also confirmed by our findings. However, by using latent trajectory analysis to identify groups of individuals who show similar developmental trajectories over time, we found that a large majority of participants actually maintain their perceived health status during retirement transition and smaller subgroups of people show improvement or decline in perceived health (50). Those whose health perception declined during the retirement transition, had lower occupational status, physically strenuous work, and job strain. Overall, our findings suggest that people with physically or psychosocially demanding work may improve their psychological wellbeing after retirement, but this is not necessarily reflected in their health perception. Our future work with the extended follow-up will reveal more about the long-term development of health, morbidity as well as physical and cognitive functioning in the post-retirement years.



For peer review only - http://bmjopen.bmj.com/site/about/guidelines.xhtml

*Predictors of extended employment*

By using the information about timing of retirement, we have examined how factors related to health, work, as well as family and social relations predict retirement timing. We have observed that those who have better health and work ability (51), good worktime control (52), and full-time working spouse (53) are more likely to extend their employment beyond the pensionable age compared to those who retire at their state pension age or earlier. We have also shown that men are more likely to extend their employment than women, and this is largely explained by men having the above mentioned characteristics more often (54). We have also compared health development among those extending their employment to those who retire early or at their pensionable age and found that voluntary extension of the working career beyond pensionable age does not have either positive or negative effects on health and physical functioning among older workers (55). Our results suggest that it would be important to develop and implement practices that support extended and flexible working life participation among older workers.

The complete list of publications can be found at the FIREA study website (https://sites.utu.fi/firea/en/publications/).

**Future plans**

The second phase of the FIREA study will take place in 2023–2025, when participants reach the age of 70 years. All participants who have participated to at least one survey, accelerometer measurement, or clinical examination visit will be invited to the respective follow-up measurements. New information sheet and informed consent will be provided to the participants.

The questionnaire survey will include the same questions as previously, except questions related to work and retirement are removed and new questions


For peer review only - http://bmjopen.bmj.com/site/about/guidelines.xhtml

relevant for the age group will be included, for example weight history, chronotype, muscle strengthening and balance improving activities, functional ability, fear of falling, mental wellbeing, social relationship, loneliness, financial situation and working while retired. Activity sub-study will be conducted using similar protocol and devices as in the previous study waves. The clinical sub-study will also consist of the same measurements as in the previous study waves with few additions such as carotid artery ultrasound measurement, Montreal Cognitive Assessment (MoCA) test and the 6-minute walk test, which will replace the submaximal bicycle ergometer test. The long-term aim is to conduct subsequent follow-ups every five years and follow participants into old age.In the upcoming years and with the new data the focus of the FIREA study will shift more towards examining the determinants and development of health and functioning with an advancing age. It is of interest to study for example which factors associate maintenance of good physical and cognitive function after retirement.

**Collaboration**

Further information about the FIREA study can be found from the study website https://sites.utu.fi/firea/en/. The FIREA study welcomes research collaboration proposals that fall within the general aims of the project. We follow the EU general data protection regulation (679/2016) and the Finnish Data Protection Act (1050/2018) in data management and use. Anonymized partial datasets of the FIREA study are available by application with bona fide researchers with an established scientific record and bona fide organizations. For additional details, please contact principal investigator of the FIREA study Sari Stenholm.

For peer review only

## Data availability statement

Anonymized partial datasets of the FIREA study may be shared on a case-by-case basis upon request for bona fide researchers with an established scientific record and bona fide organizations. Data sharing outside the group is done in collaboration with FIREA group and requires a data-sharing agreement. Investigators can submit an expression of interest to FIREA research group at University of Turku, Finland (firea@utu.fi).

For peer review only

## Ethics approval

The FIREA study has been approved by the Ethics Committee of Hospital District of Southwest Finland (84/1801/2014). All FIREA participants have given written informed consent to participate in the study.

## Author contributions

SS and JV designed the cohort study; SS and KS drafted the manuscript; KS and JP performed the data analysis. SS, KS, TL, SM, AP, JP and JV critically revised the manuscript and approved the final manuscript.

## Funding

The FIREA study has been financially supported by Academy of Finland [264944, 286294, 294154, 319246 and 332030]; Ministry of Education and Culture; Hospital District of Southwest Finland, Finnish State Grants for Clinical Research; Finnish Work Environment Fund [118060]; Juho Vainio Foundation; Signe and Ane



For peer review only - http://bmjopen.bmj.com/site/about/guidelines.xhtml

Gyllenberg Foundation, Päivikki and Sakari Sohlberg Foundation, and The Finnish Foundation for Cardiovascular Research.

**Acknowledgements**

The FIREA study warmly thanks the study participants for their continuous commitment to the study. The research assistant Kati Pyykkö, study nurses Hanna Pynnönen and Minna Hyppönen, data manager Jesse Pasanen and other assisting personnel are acknowledged for their invaluable effort in the project.

**Conflict of interest**

None declared.

For peer review only

## References


1. Ekerdt DJ. Frontiers of research on work and retirement. J Gerontol B Psychol Sci Soc Sci. 2010;65B(1):69-80.

2. Behncke S. Does retirement trigger ill health? Health Economics. 2012;21(3):282-300.

3. Rohwedder S, Willis RJ. Mental Retirement. The journal of economic perspectives : a journal of the American Economic Association. 2010;24(1):119-38.

4. Calvo E, Sarkisian N, Tamborini CR. Causal effects of retirement timing on subjective physical and emotional health. J Gerontol B Psychol Sci Soc Sci. 2013;68(1):73-84.

5. Sonnega A, Faul JD, Ofstedal MB, Langa KM, Phillips JW, Weir DR. Cohort Profile: the Health and Retirement Study (HRS). Int J Epidemiol. 2014;43(2):576-85.

6. Steptoe A, Breeze E, Banks J, Nazroo J. Cohort profile: the English longitudinal study of ageing. Int J Epidemiol. 2013;42(6):1640-8.

7. Börsch-Supan A, Brandt M, Hunkler C, Kneip T, Korbmacher J, Malter F, et al. Data Resource Profile: the Survey of Health, Ageing and Retirement in Europe (SHARE). Int J Epidemiol. 2013;42(4):992-1001.

8. Sjösten N, Kivimäki M, Singh-Manoux A, Ferrie JE, Goldberg M, Zins M, et al. Change in physical activity and weight in relation to retirement: the French GAZEL Cohort Study. BMJ open. 2012;2:e000522-2011-. Print 2012.

9. Westerlund H, Kivimäki M, Singh-Manoux A, Melchior M, Ferrie JE, Pentti J, et al. Self-rated health before and after retirement in France (GAZEL): a cohort study. Lancet. 2009;374(9705):1889-96.

10. Westerlund H, Vahtera J, Ferrie JE, Singh-Manoux A, Pentti J, Melchior M, et al. Effect of retirement on major chronic conditions and fatigue: French GAZEL occupational cohort study. BMJ. 2010;341:c6149.

11. Vahtera J, Westerlund H, Hall M, Sjosten N, Kivimaki M, Sal OP, et al. Effect of retirement on sleep disturbances: the GAZEL prospective cohort study. Sleep. 2009;32(11):1459-66.

12. Barnett I, van Sluijs EM, Ogilvie D. Physical activity and transitioning to retirement: a systematic review. American Journal of Preventive Medicine. 2012;43(3):329-36.

13. Keva. Pension reform 2017. Retrieved from https://www.keva.fi/en/pensions/information-about-pensions/pension-reform-2017/. October 7, 2023. 2018.

14. Pulakka A, Leskinen T, Koster A, Pentti J, Vahtera J, Stenholm S. Daily physical activity patterns among aging workers: the Finnish Retirement and Aging Study (FIREA). Occup Environ Med. 2019;76(1):33-9.


For peer review only

15. Suorsa K, Pulakka A, Leskinen T, Heinonen I, Heinonen OJ, Pentti J, et al. Objectively Measured Sedentary Time Before and After Transition to Retirement: The Finnish Retirement and Aging Study. The Journals of Gerontology: Series A. 2019;75(9):1737-43.
16. Myllyntausta S, Pulakka A, Salo P, Kronholm E, Pentti J, Vahtera J, et al. Changes in accelerometer-measured sleep during the transition to retirement: the Finnish Retirement and Aging (FIREA) study. Sleep. 2020;43(7):zsz318.
17. Suorsa K, Leskinen T, Pasanen J, Pulakka A, Myllyntausta S, Pentti J, et al. Changes in the 24-h movement behaviors during the transition to retirement: compositional data analysis. Int J Behav Nutr Phys Act. 2022;19(1):121.
18. Greff MJE, Levine JM, Abuzgaia AM, Elzagallaai AA, Rieder MJ, van Uum SHM. Hair cortisol analysis: An update on methodological considerations and clinical applications. Clinical biochemistry. 2019;63:1-9.
19. Stenholm S, Pulakka A, Leskinen T, Pentti J, Heinonen OJ, Koster A, et al. Daily Physical Activity Patterns and Their Association With Health-Related Physical Fitness Among Aging Workers-The Finnish Retirement and Aging Study. J Gerontol A Biol Sci Med Sci. 2021;76(7):1242-50.
20. Karelius S, Vahtera J, Suorsa K, Heinonen OJ, Pentti J, Niiranen TJ, et al. Changes in ambulatory blood pressure during the transition to retirement. J Hypertens. 2023;41(1):187-93.
21. Guralnik JM, Ferrucci L, Simonsick EM, Salive ME, Wallace RB. Lower-extremity function in persons over the age of 70 years as a predictor of subsequent disability. New England Journal of Medicine. 1995;332:556-61.
22. Guralnik JM, Simonsick EM, Ferrucci L, Glynn RJ, Berkman LF, Blazer DG, et al. A Short Physical Performance Battery Assessing Lower Extremity Function: Association With Self-Reported Disability and Prediction of Mortality and Nursing Home Admission. J Gerontol. 1994;49(2):M85-M94.
23. Suni JH, Oja P, Miilunpalo SI, Pasanen ME, Vuori IM, Bos K. Health-related fitness test battery for adults: associations with perceived health, mobility, and back function and symptoms. Arch Phys Med Rehabil. 1998;79(5):559-69.
24. Studenski SA, Peters KW, Alley DE, Cawthon PM, McLean RR, Harris TB, et al. The FNIH sarcopenia project: rationale, study description, conference recommendations, and final estimates. J Gerontol A Biol Sci Med Sci. 2014;69(5):547-58.
25. Teräs T, Rovio S, Spira AP, Myllyntausta S, Pulakka A, Vahtera J, et al. Associations of accelerometer-based sleep duration and self-reported sleep difficulties with cognitive function in late mid-life: the Finnish Retirement and Aging Study. Sleep Med. 2020;68:42-9.
26. Reitan RM. Trail Making Test: Manual for Administration and Scoring. Tuscon, AZ: Reitan Neuropsychology Laboratory; 1992. Contract No.: Report.


For peer review only - http://bmjopen.bmj.com/site/about/guidelines.xhtml

27. Morris JC, Heyman A, Mohs RC, Hughes JP, van Belle G, Fillenbaum G, et al. The consortium to establish a registry for Alzheimer´s disease (CERAD). Part 1. Clinical and neuropsychological assessement of Alzheimer´s disease. Neurology. 1989;39:1159-65.
28. Beck AT, Ward CH, Mendelson M, Mock J, Erbaugh J. An inventory for measuring depression. Arch Gen Psychiatry. 1961;4:561-71.
29. Skotte J, Korshoj M, Kristiansen J, Hanisch C, Holtermann A. Detection of physical activity types using triaxial accelerometers. J Phys Act Health. 2014;11(1):76-84.
30. Stemland I, Ingebrigtsen J, Christiansen CS, Jensen BR, Hanisch C, Skotte J, et al. Validity of the Acti4 method for detection of physical activity types in free-living settings: comparison with video analysis. Ergonomics. 2015;58(6):953-65.
31. Suorsa K, Pulakka A, Leskinen T, Pentti J, Holtermann A, Heinonen OJ, et al. Comparison of Sedentary Time Between Thigh-Worn and Wrist-Worn Accelerometers. Journal for the Measurement of Physical Behaviour. 2020;3(3):234-43.
32. Brondeel R, Wasfi R, Perchoux C, Chaix B, Gerber P, Gauvin L, et al. Is older adults' physical activity during transport compensated during other activities? Comparing 4 study cohorts using GPS and accelerometer data. Journal of Transport & Health. 2019;12:229-36.
33. Pasanen S, Halonen JI, Pulakka A, Kestens Y, Thierry B, Brondeel R, et al. Contexts of sedentary time and physical activity among ageing workers and recent retirees: cross-sectional GPS and accelerometer study. BMJ Open. 2021;11(5):e042600.
34. Pasanen S, Halonen JI, Suorsa K, Leskinen T, Kestens Y, Thierry B, et al. Does work-related and commuting physical activity predict changes in physical activity and sedentary behavior during the transition to retirement? GPS and accelerometer study. Health Place. 2023;81:103025.
35. Suorsa K, Mattila VM, Leskinen T, Heinonen OJ, Pentti J, Vahtera J, et al. Work ability and physical fitness among aging workers: the Finnish Retirement and Aging Study. Eur J Ageing. 2022;19(4):1301-10.
36. American College of Sports Medicine. ACSM's Guidelines for Exercise Testing and Prescription. 9th ed. Baltimore: Wolters Kluwer, Lippincott Williams & Wilkins; 2014.
37. Lindroos AS, Johansson JK, Puukka PJ, Kantola I, Salomaa V, Juhanoja EP, et al. The association between home vs. ambulatory night-time blood pressure and end-organ damage in the general population. J Hypertens. 2016;34(9):1730-7.
38. Finland S. Grid Database 2023 [11.1.2023]. Available from: https://www.stat.fi/tup/ruututietokanta/index_en.html.
39. Pulakka A, Leskinen T, Suorsa K, Pentti J, Halonen JI, Vahtera J, et al. Physical Activity across Retirement Transition by Occupation and Mode of Commute. Med Sci Sports Exerc. 2020.

For peer review only

40. Suorsa K, Pulakka A, Leskinen T, Pentti J, Vahtera J, Stenholm S. Changes in prolonged sedentary behaviour across the transition to retirement. Occupational and Environmental Medicine. 2021;78(6):409-12.
41. Myllyntausta S, Salo P, Kronholm E, Pentti J, Oksanen T, Kivimäki M, et al. Does removal of work stress explain improved sleep following retirement? The Finnish Retirement and Aging study. Sleep. 2019;42(8):zsz109.
42. 2018 Physical Activity Guidelines Advisory Committee. 2018 Physical Activity Guidelines Advisory Committee Scientific Report. U.S. Department of Health and Human Services; 2018.
43. Pahor M, Guralnik JM, Ambrosius WT, Blair S, Bonds DE, Church TS, et al. Effect of Structured Physical Activity on Prevention of Major Mobility Disability in Older Adults: The LIFE Study Randomized Clinical Trial. JAMA. 2014;311(23):2387-96.
44. Singh B, Olds T, Curtis R, Dumuid D, Virgara R, Watson A, et al. Effectiveness of physical activity interventions for improving depression, anxiety and distress: an overview of systematic reviews. British Journal of Sports Medicine. 2023:bjsports-2022-106195.
45. Lahdenperä M, Virtanen M, Myllyntausta S, Pentti J, Vahtera J, Stenholm S. Psychological Distress During the Retirement Transition and the Role of Psychosocial Working Conditions and Social Living Environment. J Gerontol B Psychol Sci Soc Sci. 2022;77(1):135-48.
46. KC P, Virtanen M, Törmälehto S, Myllyntausta S, Pentti J, Vahtera J, et al. Changes in life satisfaction during the transition to retirement: findings from the FIREA cohort study. Eur J Ageing. 2022;19(4):1587-99.
47. van der Heide I, van Rijn RM, Robroek SJ, Burdorf A, Proper KI. Is retirement good for your health? A systematic review of longitudinal studies. BMC Public Health. 2013;13:1180.
48. Syse A, Veenstra M, Furunes T, Mykletun RJ, Solem PE. Changes in Health and Health Behavior Associated With Retirement. J Aging Health. 2017;29(1):99-127.
49. Oshio T, Kan M. The dynamic impact of retirement on health: Evidence from a nationwide ten-year panel survey in Japan. Prev Med. 2017;100:287-93.
50. Stenholm S, Virtanen M, Pentti J, Oksanen T, Kivimaki M, Vahtera J. Trajectories of self-rated health before and after retirement: evidence from two cohort studies. Occup Environ Med. 2020;77(2):70-6.
51. KC P, Virtanen M, Kivimäki M, Ervasti J, Pentti J, Vahtera J, et al. Trajectories of work ability from mid-life to pensionable age and their association with retirement timing. Journal of Epidemiology and Community Health. 2021;75(11):1098-103.
52. Virtanen M, Myllyntausta S, Kauppi M, Kivimäki M, Pentti J, Ervasti J, et al. Trajectories of Worktime Control From Midlife to Retirement and Working Beyond Retirement Age. Work, Aging and Retirement. 2021;8(3):273-81.

For peer review only

53. Kauppi M, Prakash KC, Virtanen M, Pentti J, Aalto V, Oksanen T, et al. Social relationships as predictors of extended employment beyond the pensionable age: a cohort study. Eur J Ageing. 2021;18(4):491-501.
54. Myllyntausta S, Virtanen M, Pentti J, Kivimäki M, Vahtera J, Stenholm S. Why do men extend their employment beyond pensionable age more often than women? a cohort study. European Journal of Ageing. 2022;19(3):599-608.
55. KC P, Virtanen M, Pentti J, Kivimäki M, Vahtera J, Stenholm S. Does working beyond the statutory retirement age have an impact on health and functional capacity? The Finnish Retirement and Aging cohort study. Occup Environ Med. 2020.

For peer review only

**Figure 1. Flow of the Finnish Retirement and Aging study (FIREA).**

**Figure 2. Schematic overview of the data collection in the Finnish Retirement and Aging study (FIREA).**

Bar color intensity indicates the working status of the participants. Light color = full time working, dark color = retired.

For peer review only

**Table 1. Baseline characteristics of the Finnish Retirement and Aging study (FIREA) participants and non-participants.**

| | Survey cohort (n=6,783) | Activity sub-study (n=908) | Clinical sub-study (n=290) | Non-participants (n=3,846) |
|---|---|---|---|---|
| Age (years), mean (SD) | 62.7 (1.2) | 62.4 (1.1) | 62.4 (1.0) | 62.7 (1.2) |
| Female, % | 82 | 85 | 83 | 75 |
| Occupational group, % | | | | |
| Manual | 40 | 33 | 31 | 45 |
| Lower non-manual | 30 | 29 | 34 | 28 |
| Upper non-manual | 30 | 38 | 35 | 26 |
| Married / cohabiting, % | 71 | 71 | 72 | |
| Physical activity (MET-hours per week) [a], mean (SD) | 23.6 (20.2) | 24.8 (20.0) | 28.8 (21.7) | |
| Body Mass Index (kg/m$^2$) [a], mean (SD) | 26.9 (4.6) | 26.6 (4.7) | 26.0 (4.0) | |
| Current smoking, % | 11 | 6 | 5 | |
| Alcohol risk use [b], % | 3 | 2 | 3 | |
| Good or rather good self-rated health [c], % | 73 | 77 | 82 | |



For peer review only - http://bmjopen.bmj.com/site/about/guidelines.xhtml

*Notes:* [a] Self-reported information; [b] Alcohol risk use: >24 units/week for men and >16 units/week for women. [c] Self-rated health was assessed by asking participants to rate their overall health status on a 5-point scale (1=good, 2=rather good, 3=average, 4=rather poor, 5=poor). MET = metabolic equivalent.

For peer review only

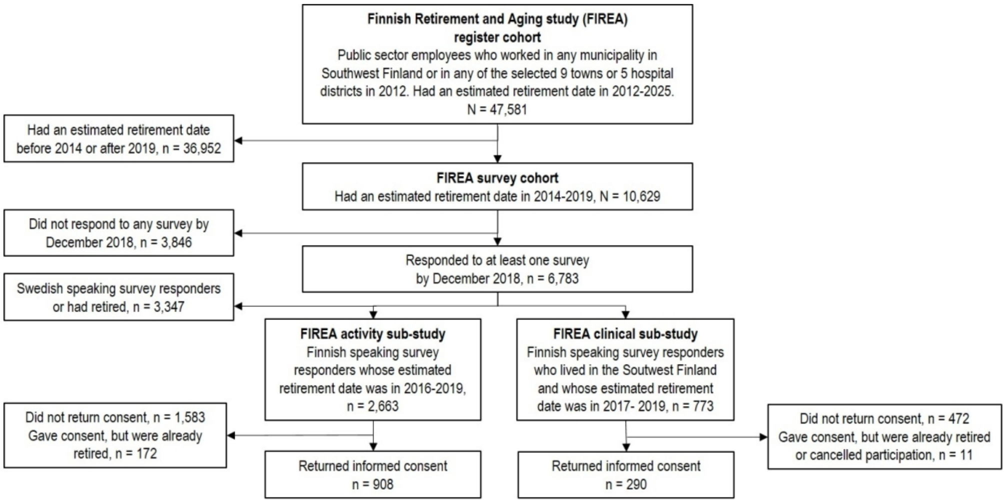


Figure 1. Flow of the Finnish Retirement and Aging study (FIREA).

257x128mm (150 x 150 DPI)

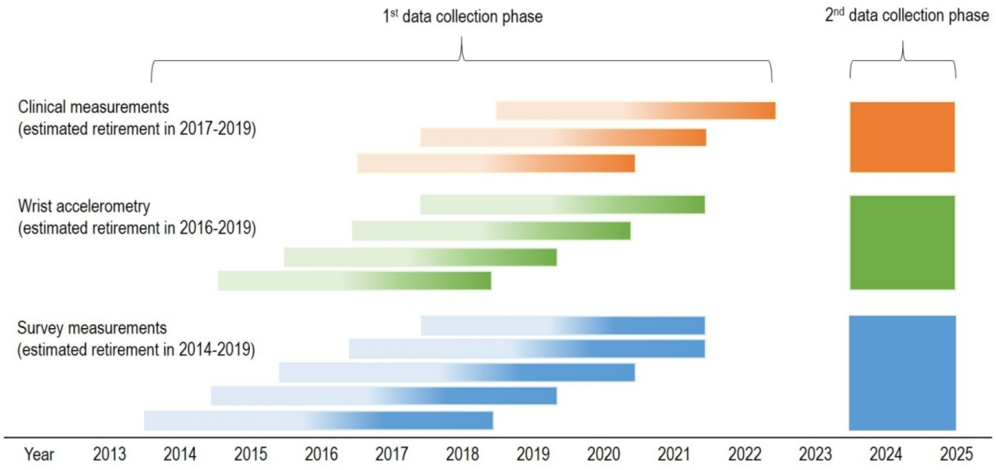


Figure 2. Schematic overview of the data collection in the Finnish Retirement and Aging study (FIREA).

Bar color intensity indicates the working status of the participants. Light color = full time working, dark color = retired.

270x133mm (150 x 150 DPI)

**Supplementary Table 1. Overview of the data collection methods in the Finnish Retirement and Aging study.**

| | |
|---|---|
| **Survey questionnaire** | |
| Sociodemographic characteristics | Gender, marital status |
| Health and wellbeing | Self-rated health, chronic conditions, vision, hearing, pain [1], height, weight, physical functioning (Short Form Health Survey (SF-36)) [1], falls, subjective memory complaints, depression [2,3], psychological wellbeing (General Health Questionnaire (GHQ)-12) [4], optimism and pessimism (Life Orientation test) [5], life satisfaction [6], sexual health (Female Sexual Function Index)[7], relationship happiness |
| Health behaviors | Sleep duration [8], sleep difficulties (Jenkins Sleep Problem Scale) [9], daytime tiredness [8], napping, sitting [10], leisure and commuting physical activity [11], smoking, alcohol consumption, diet (rhythm and content) |
| Social and living environment | Social networks [12], social participation [13], family caregiving, negative life events, neighborhood cohesion [14], health care use |
| Work and retirement | Workability [15], worktime mode, psychosocial stressors (job strain, work demands and control [16], worktime control [17], effort-reward imbalance [18], organizational justice [19]), physical work stressors (strenuous work, awkward working positions, repetitive work), plans for retirement timing, actual retirement date |
| | |
| **Device-based movement behavior measures** | |
| ActiGraph accelerometer (wActiSleep-BT and wGT3X-BT), wrist-worn | Total amount of physical activity as vector magnitude counts per minute (VM CPM) [20]. Time spent sedentary, in light physical activity, in moderate-to-vigorous physical activity, bouts (number and duration) of sedentary time, light physical activity and moderate-to-vigorous physical activity [21,22], sleep duration [22,23], sleep efficiency [23], bedtime and awakening time, ambient light [24] |
| Axivity accelerometer (AX3), thigh-worn | Time spent lying, sitting, standing, moving, walking slow, walking fast, stair walking, running, cycling and other physical activity. Number of steps and sit-to-stand transitions. Duration of lying, sitting and standing bouts [25,26] |



For peer review only - http://bmjopen.bmj.com/site/about/guidelines.xhtml

| | |
|---|---|
| Global Positioning System and accelerometer device (SenseDoc V.2.0), waist-worn | Time spent sedentary, in light physical activity and in moderate-to-vigorous physical activity in different locations and during travel [27-29]. |
| | |
| **Clinical measurements** | |
| Blood samples | Fasting blood sample were drawn by venipuncture. All blood samples were aliquoted at the same day and then stored at -80°C. At the moment some samples have been analyzed at the laboratory of Turku University Hospital, Finland. Plasma cholesterol (total, low-density lipoprotein cholesterol, high-density lipoprotein cholesterol) and triglycerides were determined by enzymatic colorimetric tests (Cobas 8000 c702, Roche Diagnostics). C-reactive protein was determined by nephelometric method (BN ProSpex/Atellica Neph, Siemens Healthineers). Fasting glucose was determined by enzymatic reference method with hexokinase and fasting insulin was measured by ECLIA/Sandwich principle (Cobas 8000 e801, Roche Diagnostics). |
| Hair sample | A strand of hair was cut from a standardized area of the posterior vertex region of the head as close to the scalp as possible [30]. Hair samples were stored in foil in a dry place protected from light. Hair cortisol and cortisone were analyzed later using mass spectrometry (Technical University of Dresden, Germany). |
| Anthropometry and body composition | Weight, height, body mass index, waist circumference, body composition (Inbody 720, Biospace Co., Seoul, Korea) [31]. |
| Cardiovascular measurements | Systolic and diastolic blood pressure from both arms and ankles (Microlife WatchBP Office Central, Microlife AG, Widnau, Switzerland) [32], ankle brachial index, 24-h ambulatory blood pressure (Microlife WatchBP O3 Monitor, Microlife AG, Widnau, Switzerland) [32], carotid-femoral Pulse Wave Velocity (SphygmoCor PVx with MM3 electronic module and Millar tonometer) [33]. |
| Physical fitness | VO2peak measured with indirect submaximal bicycle ergometer test with Ergoselect 100 K (Ergoline, Bitz, Germany) according to the American College of Sports Medicine guidelines [31,34], modified push-up test [31,35], sit-up test [35]. |



For peer review only - http://bmjopen.bmj.com/site/about/guidelines.xhtml

| | |
|---|---|
| Physical functioning | Maximal and normal walking speed over 4 meters [36], Short Physical Performance Battery [37], 1-leg standing balance [38], hand grip strength from the dominant hand (Jamar dynamometer). |
| Cognitive functioning | Cambridge Neuropsychological Test Automated Battery (CANTAB®), computerized test battery covering multiple cognitive domains including learning and memory, working memory, information processing, and reaction time [39], Trail Making Test [40], Mini Mental State Examination and verbal recall from the Consortium to Establish a Registry for Alzheimer's Disease (CERAD) [41]. |
| Self-reported medication use | Anatomical Therapeutic Chemical (ATC) Classification codes for each reported drug. |
| | |
| **Register data** | |
| Sociodemographic characteristics | Date of birth, sex, occupational code, employment history (Pension insurance institute Keva) |
| National health registers | Primary diagnosis and date of hospital discharge from years 1987-2019 (Care Register for Health Care maintained by the Finnish Institute for Health and Welfare); medication purchase, special reimbursement, rehabilitation, sick-leave days from years 1994-2019 (Registers of the Social Insurance Institution of Finland); type of cancer, date of diagnosis years 1953-2019 (Cancer register) |
| Occupational injuries | Types and causes of workplace and commuting injuries from years 2000-2019 (the Federation of Accident Insurance Institutions) |
| Residential environment characteristics | Home address; socioeconomic disadvantage: median household income, low educational attainment (percentage of people over 18 years old with low education) and unemployment rate from each 250x250m square [42]; Greenness in the neighbourhood calculated as Normalised Difference Vegetation Index (NDVI) for each map grip from a satellite image composite using Google Earth Engine [42]. Mean, maximum and minimum temperature and humidity data from >500 weather stations over Finland interpolated to a spatial resolution of 1x1km$^2$ to measure continuous daily gridded time series of daily temperature. |

3

For peer review only

## References


1. Hayes R, Sherbourne C, Mazel R. The RAND 36-item Health Survey 1.0. *Health Economics* 1993; **2**: 217–27.
2. Arroll B, Khin N, Kerse N. Screening for depression in primary care with two verbally asked questions: cross sectional study. *Bmj* 2003; **327**(7424): 1144-6.
3. Beck AT, Ward CH, Mendelson M, Mock J, Erbaugh J. An inventory for measuring depression. *Arch Gen Psychiatry* 1961; **4**: 561-71.
4. Goldberg DP. The detection of psychiatric illness by questionnaire. Oxford: Oxford university press; 1972.
5. Kronström K, Karlsson H, Nabi H, et al. Optimism and pessimism as predictors of work disability with a diagnosis of depression: a prospective cohort study of onset and recovery. *J Affect Disord* 2011; **130**(1-2): 294-9.
6. Koivumaa-Honkanen H, Honkanen R, Viinamäki H, Heikkilä K, Kaprio J, Koskenvuo M. Self-reported life satisfaction and 20-year mortality in healthy Finnish adults. *Am J Epidemiol* 2000; **152**(10): 983-91.
7. Rosen R, Brown C, Heiman J, et al. The Female Sexual Function Index (FSFI): a multidimensional self-report instrument for the assessment of female sexual function. *Journal of sex & marital therapy* 2000; **26**(2): 191-208.
8. Myllyntausta S, Salo P, Kronholm E, et al. Does removal of work stress explain improved sleep following retirement? The Finnish Retirement and Aging study. *Sleep* 2019; **42**(8): zsz109.
9. Jenkins CD, Stanton BA, Niemcryk SJ, Rose RM. A scale for the estimation of sleep problems in clinical research. *Journal of clinical epidemiology* 1988; **41**(4): 313-21.
10. Leskinen T, Pulakka A, Heinonen OJ, et al. Changes in non-occupational sedentary behaviours across the retirement transition: the Finnish Retirement and Aging (FIREA) study. *J Epidemiol Community Health* 2018; **72**(8): 695-701.
11. Kujala UM, Kaprio J, Sarna S, Koskenvuo M. Relationship of leisure-time physical activity and mortality: the Finnish twin cohort. *Journal of American Medical Association* 1998; **279**(6): 440-4.
12. Antonucci T. Measuring social support networks: Hierarchical mapping technique. *Generations: Journal of the American Society on Aging* 1986; **10**(4): 10-2.
13. Kauppi M, Prakash KC, Virtanen M, et al. Social relationships as predictors of extended employment beyond the pensionable age: a cohort study. *Eur J Ageing* 2021; **18**(4): 491-501.
14. Lahdenperä M, Virtanen M, Myllyntausta S, Pentti J, Vahtera J, Stenholm S. Psychological Distress During the Retirement Transition and the Role of Psychosocial Working Conditions and Social Living Environment. *J Gerontol B Psychol Sci Soc Sci* 2022; **77**(1): 135-48.
15. Ilmarinen J, Tuomi K, Klockars M. Changes in the work ability of active employees over an 11-year period. *Scand J Work Environ Health* 1997; **23 Suppl 1**: 49-57.
16. Karasek R, Brisson C, Kawakami N, Houtman I, Bongers P, Amick B. The Job Content Questionnaire (JCQ): an instrument for internationally comparative assessments of psychosocial job characteristics. *J Occup Health Psychol* 1998; **3**(4): 322-55.
17. Ala-Mursula L, Vahtera J, Linna A, Pentti J, Kivimäki M. Employee worktime control moderates the effects of job strain and effort-reward imbalance on sickness absence: the 10-town study. *J Epidemiol Community Health* 2005; **59**(10): 851-7.
18. Siegrist J, Wege N, Pühlhofer F, Wahrendorf M. A short generic measure of work stress in the era of globalization: effort-reward imbalance. *International archives of occupational and environmental health* 2009; **82**(8): 1005-13.


For peer review only



For peer review only - http://bmjopen.bmj.com/site/about/guidelines.xhtml

19. Elovainio M, Kivimäki M, Vahtera J. Organizational justice: evidence of a new psychosocial predictor of health. *Am J Public Health* 2002; **92**(1): 105-8.
20. Pulakka A, Leskinen T, Koster A, Pentti J, Vahtera J, Stenholm S. Daily physical activity patterns among aging workers: the Finnish Retirement and Aging Study (FIREA). *Occup Environ Med* 2019; **76**(1): 33-9.
21. Suorsa K, Leskinen T, Pasanen J, et al. Changes in the 24-h movement behaviors during the transition to retirement: compositional data analysis. *Int J Behav Nutr Phys Act* 2022; **19**(1): 121.
22. Migueles JH, Rowlands AV, Huber F, Sabia S, van Hees VT. GGIR: A research Community–Driven open source R package for generating physical activity and sleep outcomes from multi-day raw accelerometer data. *Journal for the Measurement of Physical Behaviour* 2019; **2**(3): 188-96.
23. Myllyntausta S, Pulakka A, Salo P, et al. Changes in accelerometer-measured sleep during the transition to retirement: the Finnish Retirement and Aging (FIREA) study. *Sleep* 2020; **43**(7): zsz318.
24. Flynn JI, Coe DP, Larsen CA, Rider BC, Conger SA, Bassett DR, Jr. Detecting indoor and outdoor environments using the ActiGraph GT3X+ light sensor in children. *Med Sci Sports Exerc* 2014; **46**(1): 201-6.
25. Skotte J, Korshoj M, Kristiansen J, Hanisch C, Holtermann A. Detection of physical activity types using triaxial accelerometers. *J Phys Act Health* 2014; **11**(1): 76-84.
26. Stemland I, Ingebrigtsen J, Christiansen CS, et al. Validity of the Acti4 method for detection of physical activity types in free-living settings: comparison with video analysis. *Ergonomics* 2015; **58**(6): 953-65.
27. Thierry B, Chaix B, Kestens Y. Detecting activity locations from raw GPS data: a novel kernel-based algorithm. *International journal of health geographics* 2013; **12**: 14.
28. Pasanen S, Halonen JI, Pulakka A, et al. Contexts of sedentary time and physical activity among ageing workers and recent retirees: cross-sectional GPS and accelerometer study. *BMJ Open* 2021; **11**(5): e042600.
29. Pasanen S, Halonen JI, Suorsa K, et al. Does work-related and commuting physical activity predict changes in physical activity and sedentary behavior during the transition to retirement? GPS and accelerometer study. *Health Place* 2023; **81**: 103025.
30. Greff MJE, Levine JM, Abuzgaia AM, Elzagallaai AA, Rieder MJ, van Uum SHM. Hair cortisol analysis: An update on methodological considerations and clinical applications. *Clinical biochemistry* 2019; **63**: 1-9.
31. Stenholm S, Pulakka A, Leskinen T, et al. Daily Physical Activity Patterns and Their Association With Health-Related Physical Fitness Among Aging Workers-The Finnish Retirement and Aging Study. *J Gerontol A Biol Sci Med Sci* 2021; **76**(7): 1242-50.
32. Karelius S, Vahtera J, Suorsa K, et al. Changes in ambulatory blood pressure during the transition to retirement. *J Hypertens* 2023; **41**(1): 187-93.
33. Lindroos AS, Johansson JK, Puukka PJ, et al. The association between home vs. ambulatory night-time blood pressure and end-organ damage in the general population. *J Hypertens* 2016; **34**(9): 1730-7.
34. American College of Sports Medicine. ACSM's Guidelines for Exercise Testing and Prescription. 9th ed. Baltimore: Wolters Kluwer, Lippincott Williams & Wilkins; 2014.
35. Suorsa K, Mattila VM, Leskinen T, et al. Work ability and physical fitness among aging workers: the Finnish Retirement and Aging Study. *Eur J Ageing* 2022; **19**(4): 1301-10.



For peer review only - http://bmjopen.bmj.com/site/about/guidelines.xhtml

36. Guralnik JM, Ferrucci L, Simonsick EM, Salive ME, Wallace RB. Lower-extremity function in persons over the age of 70 years as a predictor of subsequent disability. *New England Journal of Medicine* 1995; **332**: 556-61.
37. Guralnik JM, Simonsick EM, Ferrucci L, et al. A Short Physical Performance Battery Assessing Lower Extremity Function: Association With Self-Reported Disability and Prediction of Mortality and Nursing Home Admission. *J Gerontol* 1994; **49**(2): M85-M94.
38. Suni JH, Oja P, Miilunpalo SI, Pasanen ME, Vuori IM, Bos K. Health-related fitness test battery for adults: associations with perceived health, mobility, and back function and symptoms. *Arch Phys Med Rehabil* 1998; **79**(5): 559-69.
39. Teräs T, Rovio S, Spira AP, et al. Associations of accelerometer-based sleep duration and self-reported sleep difficulties with cognitive function in late mid-life: the Finnish Retirement and Aging Study. *Sleep Med* 2020; **68**: 42-9.
40. Reitan RM. Trail Making Test: Manual for Administration and Scoring. Tuscon, AZ: Reitan Neuropsychology Laboratory, 1992.
41. Morris JC, Heyman A, Mohs RC, et al. The consortium to establish a registry for Alzheimer´s disease (CERAD). Part 1. Clinical and neuropsychological assessement of Alzheimer´s disease. *Neurology* 1989; **39**: 1159-65.
42. Halonen JI, Pulakka A, Pentti J, et al. Cross-sectional associations of neighbourhood socioeconomic disadvantage and greenness with accelerometer-measured leisure-time physical activity in a cohort of ageing workers. *BMJ Open* 2020; **10**(8): e038673.

For peer review only
